# Understanding Why Parents Say Yes or No to Organ Donation When Their Child Dies: Mixed‐Methods Study

**DOI:** 10.1111/jan.17091

**Published:** 2025-06-09

**Authors:** Ellie Crane, Angie Scales, Leah Mclaughlin, Roisin Hollinger, Reinout J. Mildner, Jane Noyes

**Affiliations:** ^1^ Bangor University School of Health Sciences Bangor UK; ^2^ NHS Blood and Transplant Bristol UK; ^3^ Paediatric Intensive Care Unit Birmingham Children's Hospital Birmingham UK

**Keywords:** consent, decision‐making, mixed‐methods, nursing, organ donation, paediatric, qualitative

## Abstract

**Aim:**

To explore why parents consent to or decline organ donation after their child's death and identify the factors that influence their decision‐making.

**Design:**

Mixed‐methods analysis of routinely collected quantitative and qualitative data from 594 cases in the United Kingdom between 2018 and 2024.

**Methods:**

Quantitative analysis of clinical and demographic variables of potential donors, including regression analyses examining associations with parental consent. Qualitative content analysis and frequency counts of anonymised clinical notes of parental discussions and decision‐making. Integration of quantitative and qualitative findings on similar phenomena of interest.

**Results:**

For each additional life‐year of the child, the odds of parental consent increased by 6%; the odds of consent among white families were five times higher than those of non‐white families; and the odds of consent for donation after brainstem death were 1.78 times higher than those for donation after circulatory death. Parental non‐support was influenced by the perception that organ donation prolonged or altered end‐of‐life care, cultural and religious beliefs and the need for surgery. Factors facilitating consent included altruism, creating a positive legacy, and prior knowledge of organ donation. Parents fell into four decision groups: those who immediately consented, hesitated but consented, hesitated but declined and immediately declined.

**Conclusion:**

This study provides new insights and theories about which parents are more likely to consent to paediatric organ donation. Findings highlight a range of factors that shape parental decisions and the need for approaches tailored to the varied concerns and complexities that parents face in navigating end‐of‐life care for their child. Developing specific support strategies that acknowledge contextual, religious and procedural concerns may enhance consent rates and facilitate a more bespoke family‐centred approach to paediatric organ donation and palliative care. Other countries achieve better paediatric consent rates, suggesting there is potential to further improve policy and practice, underpinned by a programme of research.

**Summary Statement:**

*What is already known*: Internationally, there is a shortage of organs for paediatric transplants, and parental consent is the limiting factor in most high‐income countries. Paediatric organ donation is unique from adults, and there is limited research into why parents consent to or decline organ donation for their child. *What this paper adds*: This analysis identified a range of interconnected factors that could positively or negatively influence parental consent decisions, with emotional, logistical, procedural and religious factors acting as critical barriers to consent. Additionally, parents were categorised into four decision‐making groups that have distinct decision‐making patterns and characteristics. *Implications for practice and policy*: These findings provide a foundation for better understanding why parents choose not to consent to organ donation for their child, which can begin to inform the development of policies and practices that better create the conditions for consent and a more family‐centred approach in paediatric end‐of‐life care.

**Implications for the Profession and/or Patient Care:**

This study highlights critical factors predicting and shaping parental decisions and indicates a need for approaches tailored to the emotional and logistical complexities that parents face. Specific support strategies that acknowledge contextual, religious and procedural concerns may facilitate a more bespoke family‐centred approach to paediatric end‐of‐life care and enhance organ donation consent rates.

**Impact:**

*What problem did the study address?* Internationally, there is a shortage of organs available for children in need of transplants. Parental consent for paediatric organ donation is a limiting factor in most high‐income countries. The emotive nature of a child's death makes paediatric organ donation complex and unique, and there is limited research on the factors influencing parental decision‐making and the reasons parents choose to consent to or decline organ donation after their child's death. *What were the main findings?* We identified a range of interconnected factors that could positively or negatively influence parental consent decisions, with emotional, logistical, procedural and religious factors acting as critical barriers to consent. For the first time, parents have been categorised into four decision‐making groups that have distinct decision‐making patterns and characteristics. A novel and prevalent factor that negatively impacted consent was that organ donation prolonged or altered end‐of‐life care, often due to organ donation being raised after plans had been made for end‐of‐life care. This highlights the need for health systems to adopt proactive and timely approaches to integrating paediatric organ donation with care pathways. This study also identified that parents were 1.78 times more likely to consent to organ donation via a donation by brain death pathway compared to a donation by circulatory death pathway. *Where and on whom will the research have an impact?* This is the largest study of paediatric organ donation outside of the United States (where the healthcare system is predominantly for‐profit and therefore a different model and context compared to the United Kingdom) and provides new insights and understandings that can assist Specialist Nurses in Organ Donation, clinical teams and palliative care specialists in tailoring their approach to end‐of‐life care to achieve the best consent outcome for each child, their parents and wider family. Findings provide a valuable foundation for the development of future research and developments in policy and practice in the United Kingdom and countries with similar health systems. Findings also include generalisable insights that can be utilised internationally, irrespective of the health system.

**Reporting Methods:**

This study adhered to the Good Reporting of a Mixed‐Methods Study (GRAMMS) reporting standards.

**Patient or Public Contribution:**

Patients or the public were not involved in this study.

**Summary**

- What does this paper contribute to the wider global clinical community?
  ○ This paper makes a valuable new contribution to understanding parental decision‐making when considering organ donation for a child, including predictors of consent and key factors facilitating or hindering consent, offering generalisable implications for practice and policy at an international level.
  ○ The factors influencing parental decision‐making in paediatric organ donation are varied, nuanced, and multifaceted, highlighting the need for tailored and targeted approaches that address the specific emotional, logistical, and procedural challenges faced by different groups of parents.
  ○ Parents for the first time have been categorised into four distinct decision‐making groups, which provide a theoretical framework for informing future research, policy and practice.

## 1 Introduction

Paediatric organ transplantation is a well‐established lifesaving procedure for children with end‐stage organ failure. However, in most high‐income countries, the shortage of donor organs severely limits the availability of organ transplantation for children under 18 years old (Paediatric Subgroup of the National Organ Donation Committee 2019; George et al. 2022). This scarcity is especially acute in paediatrics due to the relative rarity of child deaths and the need for size‐matched organs, which reduces the pool of potential donors when compared to adults (George et al. 2022).

### 1.1 Background

In the United Kingdom, many children die each year while awaiting an organ transplant, and since 2018, the transplant waiting list has grown from 130 children to 238 in 2023 (Paediatric Subgroup of the National Organ Donation Committee 2019; NHS Blood and Transplant, n.d.). Between 2008/2009 and 2017/2018, adult organ donation rates in the United Kingdom increased by 75%, leading to the transplant waiting list to decrease for eight consecutive years (Paediatric Subgroup of the National Organ Donation Committee 2019). This success is attributed to several factors, including the implementation of the ‘Organ Donation Taskforce’ recommendations that involved enhancement of the national organ donor register, public awareness campaigns, and improved coordination and infrastructure supporting the entire organ donation pathway (Organ Donation Taskforce 2008). However, despite substantial progress in adult organ donation, paediatric organ donation consent rates have not increased at the same rate in the United Kingdom (Paediatric Subgroup of the National Organ Donation Committee 2019). To address these challenges, the UK Paediatric and Neonatal Deceased Donation Strategic Plan was developed in 2019 (see Figure S1 for timeline of important events in paediatric organ donation in the United Kingdom) (Paediatric Subgroup of the National Organ Donation Committee 2019). This strategic plan aimed to integrate organ and tissue donation as a routine part of end‐of‐life care in paediatric and neonatal intensive care units and better address the unique challenges in donation from children. However, data from National Health Service Blood and Transplant (NHSBT), the United Kingdom body overseeing organ donation, has since reported a consistent downward trend in paediatric organ donation consent rates, decreasing from 61% in 2018/2019 to 38% in 2023/2024 (ODT Clinical—NHS Blood and Transplant 2024a). The reasons for this are not clear and there is no sign of paediatric consent rate recovery to previous levels in the post Covid‐19 pandemic period.

Over the past decade, a growing body of research has focused on adult organ donation, exploring public awareness, individual and family decision‐making, clinical and organ donation pathways, and the impact of organ donation policies and legislation on consent rates. In the United Kingdom, paediatric organ donation requires informed consent from a parent or guardian, and although a child's wishes should be honoured, their opinions on organ donation are often not known (Human Tissue Authority 2022). In the United Kingdom, family‐centred care is the model underpinning medical and nursing practice (Harrison 2009). The approach to parents by the Specialist Nurse in Organ Donation (SNOD) is delivered within this context, and often many other family members are present and contribute to the conversation. Despite the critical importance of parental/guardian consent (hereafter referred to as parental consent) in paediatric organ donation, there is a scarcity of research internationally examining parental decision‐making.

End‐of‐life care in children is generally critically under‐researched, and the emotive and tragic nature of child death and parental grief, in addition to the requirement for parental consent, makes paediatric organ donation distinct from the adult system of organ donation (Shaw et al. 2022; Brierley et al. 2015; Barrett et al. 2023). A qualitative evidence synthesis that included nine studies of varying quality and richness provided some insights into the experiences of parents faced with the decision about organ donation for their child (Overy et al. 2024). Seven themes were reported, including: concern for the child's wellbeing, family, experience of healthcare, quality and appropriateness of the organ donation approach, understanding of organ donation, values and beliefs and parallel stressors. Prior research in adult organ donation has led to the establishment of a detailed framework that integrates a broad range of psychosocial factors that influence families' decision to consent (López et al. 2018). However, a comprehensive understanding of decision‐making in paediatric organ donation remains undeveloped.

There was a clear need to develop a better understanding of the overall picture of paediatric consent and the factors that positively and negatively influence parental decision‐making, both within the United Kingdom and internationally. This new knowledge is critical to informing the further development of clinical policies and practices that consider the emotional, ethical and clinical challenges involved in paediatric organ donation.

## 2 The Study

### 2.1 Aim

To explore why parents consent to or decline organ donation after their child's death and to identify the factors that influence their decision‐making. This analysis is the first step toward further developing organ donation processes that are more cognisant and responsive to the individual circumstances, views, and concerns of parents, families, and the child.

## 3 Methods

### 3.1 Design

This study utilised a mixed‐methods approach to analysing routinely collected audit data of potential paediatric organ donors in the United Kingdom. The mixed‐methods design had aspects of both sequential and convergent parallel design types (Noyes, Booth, Moore, et al. 2019), but these design types are not useful in articulating how these data were used. In this respect, our integration strategies included both comparing and connecting the quantitative and qualitative data (Sutcliffe et al. 2024). A mixed‐methods design was selected as the audit data provided numerical data that could be subject to descriptive statistical and regression analysis and narrative data that could be subject to qualitative content analysis and frequency counts (Elo and Kyngäs 2008). Quantitative data were analysed first, then qualitative data were analysed, informed by quantitative results. Then, identified factors that created barriers or facilitators and other concepts of interest in the qualitative data were subject to frequency counts that subsequently fed into a logistical regression. Finally, there was an integration stage where quantitative and qualitative findings on similar phenomena of interest were juxtaposed. Integrating statistical analysis with more nuanced qualitative insights provided the opportunity to better understand the complexity of parental decision‐making and organ donation consent outcomes and to make the best use of available data. This study adhered to the Good Reporting of a Mixed‐Methods Study (GRAMMS) reporting standards, and a supplementary checklist detailing adherence to these standards is provided in Table S1 (O'Cathain et al. 2008).

### 3.2 Study Setting and Sampling

This study utilised data from the Potential Donor Audit, which is a dataset routinely collected for NHS Blood and Transplant and includes all potential paediatric organ donors in the United Kingdom. A potential paediatric organ donor is defined as a patient under the age of 18 years receiving care in a critical care, emergency, or other setting who meets certain clinical criteria that make organ donation possible. Paediatric patients and their parents/guardians were included in the Potential Donor Audit dataset if the child was identified and referred to NHSBT as a potential organ donor.

### 3.3 Data Collection

The Potential Donor Audit includes data from potential paediatric donors from England, Scotland, Wales and Northern Ireland, and this study used data collected from 3 November 2018, to 31 March 2024. Data for the Potential Donor Audit is collected by Specialist Nurses in Organ Donation (SNODs) for every potential paediatric organ donor. In the United Kingdom, there are different terms for the role of specialist nurses working in organ donation. In this paper, all of the specialist nursing roles will be referred to as SNODs, whose role is to obtain parental consent. Consent is considered to be obtained when the parents agree to at least one organ or tissue donation from their child.

The Potential Donor Audit included clinical and demographic information recorded on the DonorPath electronic database. This includes quantitative data such as ethnicity, age, cause of death, and whether consent was given for organ donation. Additionally, on DonorPath, SNODs record the sequence of events and summarised discussions with all parents or guardians who were approached about organ donation for their child. This is in the form of textual freehand clinical notes detailing factors such as their discussions with parents, guardians, and family members about organ donation, parental decision‐making, family circumstances, and interactions with the multi‐disciplinary healthcare team. SNOD's notes are recorded in English. There is a possibility that in some circumstances the SNODs may have called on National Health Service translators to communicate with parents whose first language was not English, but the responses would have been conveyed in English by the translator and recorded in English. The notes represent the nurse's observed/experienced perspectives and not the parents' or wider family members.

The clinical notes made by SNODs collected from 3 November 2018 to 31 March 2023 were anonymised, and relevant data extracted by two NHSBT staff members, AS and RH. The shared text included information about parental decision‐making, contextual factors, and potential factors that created barriers or facilitators to giving consent to organ donation. For the period from 1 April 2023 to 31 March 2024, sections of the original SNOD notes were extracted verbatim except for the removal of identifiable information. It was, however, possible to link the extracted text back to individual cases and their specific context, as each case had a unique identifier that was used throughout the study.

Additionally, it is also important to note that from 1 April 2023 to 31 March 2024, a new notification practice was introduced for potential paediatric organ donors that altered the timing of when some parents were approached for organ donation. As a result, some parents/guardians will have been approached earlier in comparison to previous years, which may have impacted consent rates or parent/family experiences compared to previous years.

### 3.4 Data Analysis

#### 3.4.1 Quantitative Analysis

Descriptive statistics were summarised for the following variables: sex of the child, age of the child, donation type, whether consent was given for organ donation, ethnicity of the child, cause of death, and whether a SNOD was present in the organ donation planning and discussion (known as collaborative approach). The type of donation refers to the organ donation pathway, which was either donation after circulatory death or donation after brainstem death. Donation after circulatory death refers to organ donation from patients whose death is confirmed using cardio‐respiratory criteria after an agreement has been reached to withdraw life‐sustaining treatment (ODT Clinical—NHS Blood and Transplant 2024b). Donation after brain death refers to organ donation from patients who have a diagnosis of death using neurological criteria while they remain on mechanical ventilation (ODT Clinical—NHS Blood and Transplant 2024c). Categories for cause of death were assigned by a researcher (EC), and these categories were determined in collaboration with NHSBT staff (RH and AS). The categories for cause of death were; life‐limiting illness, sudden death (e.g., trauma, drowning), sudden health event (e.g., asthma, cardiac arrest) and suicide/self‐harm. For the category ‘suicide and self‐harm’, suspected suicide or self‐harm was included, as well as known suicide or self‐harm. Categories were assigned using the recorded cause of death from the Potential Donor Audit and clinical records.

Unadjusted logistic regression analyses were conducted to examine the effect of the child's age, the child's sex, donation type, ethnicity and cause of death, on parental decisions to consent to organ donation for their child (Hosmer et al. 2013). The analyses were performed with consent as the binary outcome variable, and with the demographic/clinical characteristic as the predictor variable. Additionally, adjusted analyses were conducted to control for the child's demographic characteristics, including age, ethnicity and sex. Due to the relatively low numbers in some categories for ethnicity, ‘Asian/Asian British’, ‘Black/Black British’, ‘mixed’ and ‘other’ ethnic groups were grouped together as ‘Black, Asian and other’. Missing data were handled using listwise deletion, whereby cases missing any variables included in the regression models were excluded from the analysis. All logistic regression models were evaluated for multicollinearity, homoscedasticity and outliers. For the logistic regression model with age as a continuous predictor, the assumption of linearity was also tested. All statistical analyses were conducted using R by EC (RStudio Team 2021) and checked by a second person (MB).

#### 3.4.2 Qualitative Analysis

The results of the quantitative analysis informed potential phenomena of interest to explore. Qualitative content analysis was used to analyse anonymised SNOD clinical notes, concentrating on the factors and contexts that created barriers or facilitators to parental consent to organ donation for their child (Elo and Kyngäs 2008). The analysis strategies used did not neatly fit any single form of content analysis and instead best aligned with conceptual content analysis, as outlined in the Columbia University School of Public Health guidance, whereby the existence and frequency of concepts is examined within the text (Columbia Public Health 2025). We used the following qualitative content analysis strategies to analyse data in order to connect and compare with the quantitative data based on the seminal work by Elo and Kyngäs (2008) and the aforementioned Columbia University guidance (Columbia Public Health 2025).

Using a deductive analytical strategy, an initial coding framework was developed by all researchers (Table S2) and was informed by current research and discussions with NHSBT staff (AS, RM and RH). Data were coded line‐by‐line using the coding framework and analysed in NVivo by EC (Dhakal 2022). Then we undertook a further stage of inductive coding as we realised that some qualitative data of interest were not captured by the initial coding framework. These data were further coded by EC and categorised based on emerging factors (see subsequent coding framework in Table S2). Using the quantitative data on whether parents consented to organ donation, the factors that created barriers or facilitators were also analysed, compared and connected by whether the parents consented (or not) to assess for patterns and differences between the two groups. The number of cases in which specific factors that created barriers or facilitators were documented was counted to identify the most frequently recorded influences on parental decision‐making. The number of cases where factors creating barriers/facilitators to consent was counted for both non‐consenting and consenting parents, as many parents who ultimately consented still encountered important challenges and uncertainty in their decision‐making process. Additionally, frequency counts were not applied to all factors identified, as it was not appropriate for certain factors (e.g., clinician‐patient communication).

### 3.5 Data Integration

Data integration happened in two stages. First, we used selected quantitative results (such as consent rates and who had consented) to contextualise and add understanding to the analysis of qualitative data on factors that created barriers and facilitators to parental consent. Second, once the quantitative and qualitative analyses were completed, we identified quantitative results and qualitative findings on similar phenomena of interest and juxtaposed them in a matrix (see Table S3) to develop an integrated interpretation. Juxtaposition of different types of evidence in a matrix is a common method for integrating qualitative and quantitative evidence (see chapter 20 section 21.13 of Noyes, Booth, Cargo, et al. 2019) (Higgins et al. 2024).

### 3.6 Ethical Considerations

Ethical approval for this study was granted by the Schools of Medical & Health Sciences Ethics Committee, Bangor University (ref: 0187). Permission was also granted by NHSBT, and a data sharing agreement was signed by both parties.

### 3.7 Rigour and Reflexivity

The research team included health services researchers, a paediatric lead specialist nurse and a clinical lead (doctor) for paediatric organ donation. Potential biases were acknowledged, and data were shared and jointly interpreted in virtual and face‐to‐face team meetings until an agreed understanding was achieved from multiple perspectives. The quantitative analyses were reviewed by a second person. Quantitative and qualitative data were triangulated and integrated using an appropriate method, and additional analyses were undertaken to further contextualise and explain the qualitative findings.

## 4 Results

### 4.1 Characteristics of the Sample

From 2 November 2018, to 31 March 2024, a total of 594 families were approached about organ donation as an end‐of‐life option for their child. Overall, 292 families decided to consent to organ donation for their child, resulting in an overall consent rate of 49% (Table 1). The consent rate shows a declining trend, from 59% in 2018/2019 to 37% in 2023/2024. Overall, the mean age of paediatric potential donors was 8.8 years (SD 6.5), 41% female, and the majority were of white ethnicity (66%) (Table 2).

**TABLE 1:** Consent rates for paediatric organ donation in the United Kingdom.

Year	Number of parents approached about organ donation as an end‐of‐life option for their child	Number of parents who consented to organ donation for their child	Number of parents who did not consent to organ donation for their child	Consent rate for organ donation, %
2018/2019	46	27	19	59
2019/2020	121	70	51	58
2020/2021	100	53	47	53
2021/2022	96	50	46	52
2022/2023	118	50	68	42
2023/2024	113	42	71	37
Total	594	292	302	49

*Note:* Consent rate percentage is rounded to the nearest whole number. The annual period runs from 1 April to 31 March for each year, except for the year 2018/2019, which is a partial year from 2 November to 31 March.

**TABLE 2:** Demographic and clinical characteristics.

	All cases from 2018 to 2024 (*n* = 594)	Parents who consented to organ donation (*n* = 292)	Parents who did not consent to organ donation (*n* = 302)
Demographic characteristics			
Age of child (years)			
Mean (SD)	8.8 (6.5)	10.4 (6.2)	7.5 (6.5)
Missing	—	—	—
Sex of child			
Female	242 (41%)	120 (41%)	122 (40%)
Male	350 (59%)	172 (59%)	178 (60%)
Missing	2 (< 1%)	—	2 (< 1%)
Ethnicity of the child			
Asian or Asian British	83 (15%)	18 (6%)	65 (25%)
Black or Black British	29 (5%)	3 (1%)	26 (10%)
Mixed	25 (5%)	12 (4%)	13 (5%)
White	398 (74%)	250 (88%)	148 (58%)
Other	6 (1%)	2 (< 1%)	4 (2%)
Missing	53 (9%)	7 (2%)	46 (15%)
Clinical characteristics			
Cause of death			
Life limiting illness	174 (30%)	70 (24%)	103 (35%)
Sudden death from trauma or accident (e.g., choking, drowning)	162 (28%)	85 (30%)	77 (26%)
Sudden health event (e.g., stroke, asthma, cardiac arrest)	167 (29%)	86 (30%)	81 (27%)
Suicide or self‐harm	80 (14%)	46 (16%)	34 (12%)
Missing	11 (2%)	5 (2%)	7 (2%)
Organ donation type			
Donation after brainstem death	264 (44%)	161 (55%)	103 (34%)
Donation after circulatory death	330 (56%)	131 (45%)	199 (66%)
Missing	—	—	—
SNOD present			
SNOD present	364 (70%)	189 (75%)	175 (66%)
SNOD not present	156 (30%)	64 (25%)	92 (34%)
Missing	74 (12%)	39 (13%)	35 (12%)

*Note:* Data displayed as number of participants (%) or mean (SD). Percentages are rounded up to nearest whole number and mean (SD) is rounded up to one decimal place. Percentages for demographic and clinical characteristics are calculated from the corresponding sample size for the data available (not including missing data) in each column. Missing data percentages are calculated from the corresponding sample for each column.

Abbreviation: SNOD = Specialist Nurse in Organ Donation.

### 4.2 Quantitative Results

We found statistically significant evidence that for every 1‐year increase in the age of the child, the odds of parents giving consent for organ donation increased (adjusted odds ratio [OR]: 6%, 95% confidence interval [CI]: 3% to 10%, *p* < 0.001) (Table 3). There was statistically significant evidence that the odds of parental consent being given for organ donation were 5.03 times higher for children of white ethnicity compared to children who are not white (adjusted OR: 5.03; 95% CI: 3.27 to 7.91; *p* < 0.001). We found no evidence for an association between the sex of the child and the odds of parents giving consent in the unadjusted analysis (*p* = 0.92) and the adjusted analysis (*p* = 0.34). For the unadjusted analysis, there was moderate evidence that the odds of parental consent differed depending on the cause of death of the child. However, when adjusting for demographic variables, we found no association. When parents had the option to consent to organ donation following brainstem death, the odds of consent were 1.78 times greater compared to parents who had the option of organ donation following circulatory death (adjusted OR: 1.78, 95% CI: 1.21 to 2.63, *p* = 0.003).

**TABLE 3:** Unadjusted and adjusted logistic regression analyses.

	Unadjusted odds ratio (95% confidence interval)	*p*	Adjusted odds ratio (95% confidence interval)^a^	*p*
Age of the child				
Per 1 year	1.07 (1.05–1.10)	< 0.001	1.06 (1.03–1.10)	< 0.001
Sex of child				
Female (reference category)				
Male	0.98 (0.71–1.36)	0.92	0.832 (0.57–1.21)	0.34
Ethnicity				
Black, Asian and other (reference category)				
White	5.21 (3.41–8.12)	< 0.001	5.03 (3.27–7.91)	< 0.001
Cause of death				
Life limiting illness (reference category)				
Sudden death (e.g., trauma, choking, drowning)	1.62 (1.06–2.51)	0.028	1.00 (0.60–1.64)	0.99
Sudden health event (e.g., stroke, asthma, cardiac arrest)	1.56 (1.02–2.40)	0.041	1.04 (0.63–1.70)	0.89
Suicide or self‐harm	1.99 (1.17–3.43)	0.012	0.94 (0.48–1.86)	0.86
Donation type				
Donation after circulatory death (reference category)				
Donation after brainstem death	1.56 (1.22–2.01)	< 0.001	1.78 (1.21–2.63)	0.003

*Note:* Data displayed as odds ratio. The odds ratio is presented as the likelihood of parental consent to organ donation compared to the reference category for each variable. Odds ratios and 95% confidence intervals are rounded up to two decimal places.

^a^ Adjusted for demographic characteristics of the child (age of the child, ethnicity of the child and sex of the child).

All statistical assumptions, including multicollinearity, homoscedasticity, outliers and linearity, were met for the logistic regression models. All missing data was excluded from regression analyses by listwise deletion. For age, sex, cause of death and organ donation type, there was a low proportion of missing data (less than 1%) (Table 2). For cause of death, there was also a low proportion of missing data (2%). However, data for ethnicity of the child had a higher proportion of missing data (9%), particularly for parents who did not consent to organ donation (15%). Because data for ethnicity was often missing in non‐consent cases, listwise deletion may have introduced some bias into the regression models for ethnicity. However, given the large adjusted odds ratio (adjusted OR: 5.03; 95% CI: 3.27 to 7.91; *p* < 0.001) and the relatively modest proportion of missing data, we can be reasonably confident that ethnicity is an important predictor of parental consent.

### 4.3 Qualitative Findings

We identified a broad range of factors that created barriers or facilitators to parental consent for organ donation (briefly summarised in Table 4 and further expanded in Table S4) and assessed the frequency with which these factors occurred in the qualitative data (Table 5). Additionally, we examined how these factors were associated with different decision‐making outcomes and explored the context and patterns within the identified factors. We also analysed factors related to the healthcare system and clinician‐parent communication (briefly summarised in Table 6 and further expanded in Table S5). We compared qualitative data by year but detected no change in the factors influencing parent and family decision‐making that could explain a decline in consent.

**TABLE 4:** Factors that created barriers or facilitators to parental consent to organ donation for a child.

Theme	Findings	Quote
Factors that created barriers to parental consent for organ donation		
Organ donation prolonging or altering timings	A withdrawal of life‐supporting treatment plan was usually in place before discussing organ donation. Altering or prolonging these timings for organ donation created both emotional and logistical challenges for parents	‘Plans for the withdrawal of life‐supporting treatment had already been made prior to offering organ donation. Parents are expecting the withdrawal of life‐supporting treatment today’ ‘The parents did not want to prolong the situation any longer and wanted withdrawal of treatment’
Religion and cultural beliefs	Religious and cultural beliefs often led to non‐consent, as many parents felt organ donation conflicted with their faith and community values. It was also a factor association with mistrust and non‐acceptance	‘The family did not want the body touching after death and they required the child to remain whole to go to Allah. They specified they did not want a postmortem and would be exploring this further with the coroner or police should it be necessary. Doctor felt it was an appropriate time to bring in organ donation and sensitively explore. The male members of the family strongly declined expressing it was against their religious beliefs’
Surgery or the impact of surgery on the child's body or appearance	Strong aversion to surgery was common, with parents often using terms such as ‘butchered’ or ‘cut up’. Clinicians emphasising the dignity of the surgery and the ability to conceal incisions helped some parents reframe their perceptions	‘Approached for organ donation with an immediate negative answer from both parents stating that they did not want her to be cut up. I reassured them that we would take good care of her, and that the operation was like any other operation, performed by specialist surgeons. It would involve a clean single incision, which will be stitched and covered with a dressing that she would look the same once dressed. Family aware of the benefits for recipients but they apologised and said that although they might donate themselves, they could not do it with child’
Concerns about suffering	Parents often felt their child had suffered enough and wanted to let them go in peace without further procedures	‘Mum and dad positively regard organ donation, however declined organ donation. The family have already discussed it and they feel child has been through enough’
Non‐acceptance and acute distress	Non‐acceptance was often linked to parents being distraught, in denial, and unable to process the situation, making it hard for them to engage in discussions about end‐of‐life care and organ donation. The trauma of a child's death left many shell‐shocked and withdrawn	‘Acknowledgement from all except mum was given. Mum remained silent and expressionless. Family was processing information when Mum began screaming and rocking in the seat, visibly distressed. Mum was not accepting of the information and remained very distressed’
Anger	Parents expressed anger in various ways. Some perceived the approach as intrusive or insensitive, others felt let down by the quality of care, and some parents were angry at the situation. Among parents who consented, anger was more often directed at the situation than at the health service	‘Family angry at being offered donation, comparison to vultures. Difficulty for SNOD to speak personally to the parents’ ‘Family wished for coroner to investigate, felt the system had failed them and that their child's death was preventable. This continued throughout all end‐of‐life care decisions’
Mistrust	Some parents expressed doubts regarding test accuracy for determining neurological death, questioned healthcare motivations, and sought second opinions	‘Family believes that the patient's treatment may have been limited to facilitate organ donation’ ‘Some distrust from parents around specific tests. This appeared to come from outside advice from family members’
Fate of the organs	Parents expressed various anxieties regarding the fate of the organs	‘Mum had lots of concerns and the main concern being the possibility of organs been retrieved and not transplanted. I reiterated that she was young and healthy until this admission and that the chances of kidneys and liver being successfully transplanted were extremely high. However, I could not guarantee this with 100% certainty. Mum then apologised and said that she was going to say no as she needed 100% certainty’
Parental disagreement about organ donation	Family disagreement could impede the parents in reaching a decision, resulting in a refusal of consent	‘Understand benefits and that it was a good thing to do. Changed mind several times. Appears split in family and some persuasion from other family members’
Complex or challenging family circumstances	Complex family dynamics sometimes hindered discussions about organ donation	‘Family dynamics are extremely complicated. Other family members are in adult intensive care unit from the same accident. Siblings have yet to be informed. Parents also require separate conversations’
Staying with child after death	For donation by circulatory death, parents can spend only 5 min with their child after the heart stops before the child is taken to the operating theatre. This limited time with their child was a barrier to some parents	‘Parents unanimously in agreement that the logistics of time associated with Donation after Circulatory Death wouldn't be in keeping with what they want end‐of‐life care to look like. The idea of 5 min ‘hands‐off’ before child transferring to theatre would be further unnecessary distress and they've already been through a lot’
Translator needed	When interpreters were used, SNODs were sometimes concerned about how well parents understood the conversation	‘An interpreter used, and there are some concerns about relaying of information correctly’
Misunderstanding or confusion	Confusion centred on parents understanding of neurological determination of death and the two different organ donation pathways	‘Family understanding and expectations regarding the timings of brainstem death and withdrawal of life‐sustaining treatment were not clear’
Emotional and physical exhaustion	Most emotionally or physically exhausted parents still consented to organ donation. While exhaustion sometimes delayed discussions or complicated the process, it often did not prevent consent	‘Patient's father stated that they all feel exhausted’
Focus on specific organs	For consenting parents, some expressed preferences and concerns regarding which organs were or were not donated	‘Organ donation is something both parents want to support, but they do not want to consent to heart. They talked about the symbolism of the heart’
Parallel stressors	The SNOD notes highlighted a range of additional stressors, concerns and responsibilities that parents had to manage alongside the decision‐making process	‘Unexpected death. Dad working away and had to fly home. Mum is heavily pregnant’
Factors that create facilitators to organ donation		
Altruism	Consenting parents often expressed a desire to help other families and children	‘Although devastated the parents are adamant that they would like to help others. In fact mum asked that we ‘do everything overnight to make it possible for her to donate her organs’. Mum has said she will get comfort from this’
Something positive	Having something positive come from a very difficult and tragic event was often a reason for parents consenting	‘Parents cried with relief over the positive option’
Child wanted or would have wanted organ donation.	Many parents felt that their child would have wanted to donate, and in some instances the child had explicitly expressed or registered their wish to donate their organs	‘Brother and others in room convinced this is what child would want as he “has heart of gold”. All in agreement that he would want something positive to come out of this’
Prior knowledge or discussion of organ donation	Some parents were recorded as having raised or discussed organ donation prior to being approached	‘Parents had raised donation in a positive way and wished for more information regarding the process’
Child or family member previous or potential beneficiary of organ donation	Some families had a child or relative who was a previous or potential organ donation beneficiary. Many of these parents felt that it was the right thing to do to donate, as they themselves would have accepted a transplant	‘Their child would have potentially needed an organ transplant. The parents would have accepted a transplant, so they felt it is only right to offer to help others too’

*Note:* Quotes have been edited for grammar, readability, and to protect anonymity of the child and their parents/family. Consultant in Charge is the term used for a senior doctor in the United Kingdom that has fully completed medical training.

Abbreviation: SNOD = Specialist Nurse in Organ Donation.

**TABLE 5:** Frequency and proportion of cases where a factor was identified that created a barrier to, or facilitated parental consent to organ donation.

	Frequency and proportion of cases where factors that created barriers to parental consent for organ donation were identified		
	Parents who did not give consent to organ donation (*n* = 302)	Parents who did give consent to organ donation (*n* = 292)	Total (*n* = 594)
Factors that created barriers to parental consent to organ donation for a child			
Organ donation prolonging or altering timings for when child dies	65 (22%)	35 (12%)	100 (17%)
Religion and cultural beliefs	70 (23%)	24 (8%)	94 (16%)
Surgery, further medical operations and the impact of surgery on the child's body	42 (14%)	25 (9%)	67 (11%)
Non‐acceptance and acute distress	35 (12%)	37 (13%)	72 (12%)
Concerns about child's suffering.	48 (16%)	17 (6%)	65 (11%)
Fate of the organs	14 (5%)	19 (7%)	33 (6%)
Anger	20 (7%)	12 (4%)	32 (5%)
Physical or Emotional Exhaustion	8 (3%)	18 (6%)	26 (4%)
Parental disagreement about consenting to organ donation	16 (5%)	9 (3%)	25 (4%)
Wanting to stay with child after death	21 (7%)	8 (4%)	29 (5%)
Mistrust	20 (7%)	5 (2%)	25 (4%)
Complex or challenging family situations	12 (4%)	9 (3%)	21 (4%)
Translator needed	9 (3%)	4 (1%)	13 (2%)
Keeping the child whole	10 (3%)	2 (1%)	12 (2%)
Misunderstanding or confusion	4 (1%)	2 (1%)	6 (1%)

	Frequency and proportion of cases where factors that created facilitators to parental consent for organ donation were identified		
	Parents who did not give consent to organ donation (*n* = 302)	Parents who did give consent to organ donation (*n* = 292)	Total
Factors that created facilitators to parental consent to organ donation for a child			
Parents raised organ donation as an option or had already thought about organ donation prior to approach.	11 (3%)	65 (22%)	76 (13%)
Altruism	0	76 (26%)	76 (13%)
Something positive	0	31 (11%)	31 (5%)
Parents felt that their child would have wanted to donate	0	28 (10%)	28 (5%)
Child expressed or recorded a choice to donate	0	23 (8%)	23 (4%)
General support for organ donation (no mention for reasons why)	0	17 (6%)	17 (3%)
Child or family member previous or potential beneficiary of organ donation	0	15 (5%)	15 (3%)
Child's personality	0	12 (4%)	12 (2%)
Pride and legacy through organ donation	0	9 (3%)	9 (2%)

*Note:* Data displayed as frequency of cases (absolute numbers) and proportion of cases (%) where factors creating barriers or facilitators to parental consent for organ donation were identified, broken down by whether the parents consented. The proportion of cases where a barrier/facilitator occurred was calculated as the frequency of barrier/facilitator divided by the group size in each category.

**TABLE 6:** Factors related to the healthcare system and clinician‐parent communication that created barriers or facilitators to parental consent to organ donation for a child.

Theme	Factors that created barriers to consent	Factors that created facilitators to consent	Quotes
Parents experiences of the organ donation consent process	Some parents reported feeling overwhelmed or upset by the extensive information provided and the numerous questions asked during organ donation discussions and the consent process	Some parents who felt overwhelmed or upset, requested minimal details and engagement, specifically about surgery or specific organ. Accommodating this appeared to help parents remain supportive of their decision to consent	‘They had fatigue with questions and stated no to tissues. They did not wish for any information regarding retrieval operation’
Integration between main healthcare team and SNODs	Tensions between the main healthcare team and Specialist Nurses in Organ Donation (SNODs) obstructed the integration of organ donation into end‐of‐life care In several cases there also seemed to be a disconnect between SNODs and the main healthcare team. This appeared to be most apparent in clinical plans not being followed in collaboration with the SNODs		‘Consultant in charge declined to have SNOD present for first conversation due to ‘longstanding conflict between paediatric intensive care unit and SNODs’. Donation was raised days later by SNOD’ ‘Paediatric intensive care unit “territorial” over family. SNOD asked by the paediatric intensive care unit to leave the conversation’
Communication between clinical staff and parents	Some parents were reported to have experienced insensitive communication from the clinical team. For example, in one case, ‘family stated they felt rushed to end her life’ and another family were recorded as saying, ‘one of the consultants were cold and clinical’	Many cases showed that SNODs and clinicians communicated skilfully, responsively, and empathetically. Their reassuring approach addressed concerns and helped parents feel more comfortable with organ donation Communication from the wider team was key in fostering parental trust and reassurance. Transparent, regular, and honest communication was especially important for parents who felt inadequately informed or suspicious	‘At present child is showing some positive signs (e.g., coughs, spontaneous breaths) which Dad would like this investigating further … [later] Dad was understanding of the change of treatment plan and was grateful for our openness and honesty. Dad stated that he takes comfort knowing that we are investigating fully’
Assumptions by clinical staff about whether parents would consent to organ donation	In a few instances, clinicians assumed that parents would not want to consider organ donation, and so it would not be appropriate to ask them, thus preventing these parents from having a choice about consenting to organ donation as an end‐of‐life care option for their child	There were several examples of SNODs challenging assumptions held by other members of staff	‘Bedside nurse mentioned that she believes family would decline organ donation due to their religion (Islam). I replied cautiously and argued that this doesn't mean they would be against organ donation. However if the nursing staff and medical staff could keep organ donation in mind for future planning it would be really positive for end‐of‐life planning, enabling his family to have a choice. Bedside nurse agreed positively’
Familiarity of staff	Several parents found the involvement of numerous different staff members to be a source of added stress	For parents who consented, familiar staff was a source of comfort in navigating the organ donation process	‘Familiarity of staff was important to the parents. Father expressed no one new to enter room’ ‘Family found comfort in a SNOD remaining with their child in theatre’
Emotional reactions of clinicians	In two cases, clinicians expressed feeling emotionally conflicted about organ donation for a child under their care. This emotional conflict could have had a negative impact on the families' perception of organ donation		‘Bedside nurse felt she was keeping the child going for organ donation. The consultant in charge was also emotional as he had brought the child in by transfer’
End of life support for families		SNODs were proactive in offering different end of life options, explaining the process of organ donation, helping parents making informed decisions, and seeking to align organ donation with families wishes	‘Mum expressed desire to be present when the child's heart stops, therefore donation after circulatory death pathway was offered. Donation after brainstem death was discussed at some point, and the parents were open to this as it would enable heart donation. Ultimately parents decided to proceed with donation after circulatory death pathway so they can be with the child when their heart stops’

*Note:* Quotes have been edited for grammar, readability and to protect anonymity of the child and their parents/family. Consultant in Charge is the term used for a senior doctor that has fully completed medical training in the United Kingdom.

Abbreviation: SNOD = Specialist Nurse in Organ Donation.

### 4.4 Factors That Created Barriers to Parental Consent

Concerns about organ donation prolonging or altering the timing of the child's death were the most commonly identified factors that prevented parental consent (Table 5). Many parents and families (prior to practice change in April 2023) had already planned end‐of‐life care and the withdrawal of life support with the child's primary clinical team. The prospect of delaying this process to accommodate organ donation was often emotionally distressing, as families frequently wished for closure and an end to an incredibly challenging ordeal.

Concerns and strong emotional reactions about the surgical procedures required to remove organs and their impact on the child's body also acted as an important factor that negatively influenced parental and family decision‐making and consent. Specific aspects and processes involved in donation after circulatory death were not acceptable to all parents and families. A specific factor for many parents and family members was that donation after circulatory death restricted the time families could spend with their child after their heart stops to 5 min before they were taken to the operating theatre. Many families felt this was too short a time to spend with their child after death, too distressing, and too much of a burden to cope with. Religious and cultural beliefs were also a common factor identified in non‐consenting scenarios, with some parents and family members feeling that organ donation conflicted with their faith or community expectations and was also linked with mistrust of healthcare professionals and the wider NHS and non‐acceptance of their child's prognosis.

Our analysis highlighted that parents and family members often faced a wide range of additional stressors to manage alongside the decision‐making process. These ‘parallel stressors’ intensified an already overwhelming situation and some parents appeared reluctant to add to their burden by consenting to organ donation. Emotional factors, including non‐acceptance, distress, anger and mistrust, emerged as distinct but overlapping factors. These factors frequently created barriers that hindered parents and family members' ability to consider or engage in discussions about end‐of‐life care, including organ donation. Non‐acceptance was associated with denial and difficulty processing the situation, which made approaching discussions about end‐of‐life care and organ donation challenging. Anger manifested in various ways, including toward being approached about organ donation, dissatisfaction with care, disagreement with the prognosis, or anger at the current situation. Mistrust sometimes led parents and family members to question medical assessments, clinical motives or seek second opinions.

### 4.5 Factors That Created Facilitators to Parental Consent

The most frequent factor associated with consent was prior knowledge of organ donation, indicated by parents or family members raising the topic themselves or stating that they had already thought about or discussed organ donation. Altruistic motivations and the desire to have something positive emerge from tragedy were also common factors in parental and wider family decision‐making. Many parents were motivated by the hope that organ donation could help others and prevent other families from experiencing similar pain. Considering their child's personality or previously expressed wishes also appeared to help facilitate the parents' decision to consent.

### 4.6 Factors Related to the Healthcare System and Clinician‐Parent Communication

Some parents and family members found the organ donation discussions and consent process overwhelming due to the extensive information and numerous questions asked (Table 6, Table S5). SNODs are strongly encouraged to ask for consent for every organ and tissue separately. Even parents and family members who supported donation could find the discussions about specific organs or surgical procedures distressing. Accommodating requests for minimal engagement in the process helped some parents to remain supportive of their decision to consent. However, this suggests that the current organ donation consent process may be too emotionally burdensome for parents and families.

Skilful and empathetic SNOD and clinician communication and behaviours played an important role in reframing parental perceptions of organ donation, effectively addressing their concerns, and supporting parents and family members in navigating shock, grief and end‐of‐life care. Transparent and regular communication also facilitated trust from parents, whilst the opposite prompted frustration, distrust and suspicion. Some parents and family members also reported experiencing the approach and communication about organ donation as insensitive or as an intrusion. There was also evidence of tension, conflict and poor integration/cohesion between SNODs and the child's primary healthcare team. In several cases, SNODs documented these issues as preventing a coordinated, cohesive and optimal approach in raising organ donation with the parents and family members. Furthermore, in some instances, clinicians assumed that a family would not be interested in organ donation, often due to known factors such as religion or cultural beliefs.

### 4.7 Decision‐Making Groups

We first created a table to theorise and classify parental decision‐making processes and outcomes into four distinct groups (Table 7). In groups 2 and 3, parents seemed to make decisions weighing up the benefits and negatives of organ donation and/or their ability to cope with stressors. The majority of these parents and their family members understood and agreed with the benefits of organ donation; however, they were also faced with additional factors that created barriers to giving consent. For example, in group 3 several parents refused organ donation once they learnt that they could not be with their child for longer than 5 min after death, or that they would have to wait longer for the withdrawal of life‐supporting treatment. Many parents in this group were apologetic or felt guilty. They agreed with organ donation in principle, but other factors took priority, which led to declined consent for organ donation.

**TABLE 7:** Decision‐making groups and group characteristics of parents considering organ donation for a child.

Group	Description	Decision‐making characteristics	Quotes
1	Parents who consent to organ donation immediately and without serious concerns	Often have prior knowledge and positive attitudes regarding organ donation, altruistic or meaning‐based motives for choosing to consent, low emotional conflict, trusting	‘They feel that child would want to donate. They are aware that the process takes some time to organise, and they seemed to understand this and be comfortable with it. Family feel child would want to donate. Parents have asked about donation several times overnight and staff report they are extremely keen for this to happen’
2	Parents who have serious concerns or worries about organ donation, but choose to consent after receiving additional information, reassurance, and further consideration	Concerns or uncertainty about certain aspects of organ donation, emotional distress, parallel stressors, recognition of the benefits of organ donation	‘Mum was still concerned about suffering of her child and the timings of organ donation, but she did not want their child's life to feel wasted’
3	Parents who have serious concerns or worries about organ donation, and decide not to consent, despite receiving further information, support and considering the possibility	Concerns and uncertainty about certain aspects of organ donation, emotional distress, parallel stressors, recognition of the benefits of organ donation	‘Continued treatment not ‘fair’ on child. Feelings that things were moving fast, and they had not had time to absorb what was happening. Concerned about time to theatre after death (5 min). Acknowledge focus on living and child helping others. Stated that they felt overwhelmed by options … Felt child would want to help others. Information from oncologist suggested some risk of transmission and family felt they could never forgive themselves if this happened. Family declined mid process’
4	Parents who from the outset immediately shut down the possibility of organ donation for their child	Strong values or beliefs against organ donation (including religious), mistrusting, strong emotional reactions, no possibility for further discussion or consideration	‘Father immediately said no to organ donation … wants his little girl left alone and to remain whole. Religion and beliefs important to the family’

*Note:* Quotes have been edited for grammar, readability and to protect anonymity of the child and their parents/family.

From our data, it was not clear why there were differences in the decision‐making outcomes between groups 2 and 3. They often faced similar dilemmas but would choose different outcomes. This is likely explained by factors not included in the SNOD clinical notes and accounted for by factors such as individual heuristics and biases, contextual factors, emotional stress, or individual coping mechanisms.

### 4.8 Integration of Qualitative and Quantitative Findings

The integration of qualitative and quantitative findings is presented in Table S3, where we juxtapose statistical analyses with qualitative insights. The qualitative findings provide insights into why the odds of consent for donation after circulatory death were lower compared to donation after brainstem death. Our qualitative findings revealed that some parents and family members struggled with the logistics associated with donation after circulatory death, which did not align with their end‐of‐life wishes. For example, as reported above, with donation after circulatory death, families are restricted to a 5‐min period after the child's heart stops before the child is taken to the operating theatre. Furthermore, for donation after circulatory death, discussions about organ donation occur while the child is still alive before life‐supporting treatment is withdrawn. In contrast, for donation after brain death, the child is confirmed to be neurologically dead much earlier in the process and often before organ donation is raised. This difference may affect how parents view organ donation and the perception of their child's suffering.

There may be a link between the qualitative findings that emphasised the influential role of religious and cultural beliefs in parental decisions not to consent, and the wider family influences, and quantitative findings that found that parents from non‐white ethnic backgrounds are six times less likely to consent to organ donation. This link between these qualitative and quantitative findings is based on the theory that individuals from ethnic minority groups are more likely to be religious. It is plausible that ethnicity and religious beliefs may be interrelated in their association with parental consent outcomes. We did look for links between religion and ethnicity in the United Kingdom literature and Office for National Statistics census data and found that little data has been analysed or reported on this. Further research is needed to investigate the relationships between consent to organ donation, ethnicity, and religious beliefs.

Our initial qualitative analysis did not provide clarification of the underlying mechanism(s) regarding the lower consent rates for younger children seen in the quantitative analysis. This is possibly due to SNODs not recording detailed parent and family perspectives and also SNODs not probing or recording age‐specific observations in their notes. To further investigate, we compared the frequency and proportion of cases where a factor that created a barrier or facilitator to consent was identified, stratified across four different age groups of the child (Table S6). This analysis showed that organ donation prolonging or altering timings in end‐of‐life care was a more prevalent factor that created a barrier for parents with younger children, particularly those under the age of 1 (Table S6). For example, among non‐consenting parents with a child under 1 year old, organ donation prolonging or altering timings was a factor that created a barrier to consent for 31% of parents, compared to 16% among non‐consenting parents with a child aged 16–17 years. Additionally, parent and family aversion to surgery and the impact of surgery on the child's body was also a more prevalent factor that created a barrier with younger children. Limited time with the child after death was also a more prevalent factor that created a barrier among families with younger children. This is likely due to the higher prevalence of donation after circulatory death in younger children, which limits families to 5 min with their child before they are taken to the operating theatre.

## 5 Discussion

This is the largest analysis of routinely collected paediatric organ donation data outside of the United States, including data from 594 families, collected over 5 years by SNODs. This study highlights a broad range of critical factors shaping parents' decisions about whether to consent to organ donation for their child. This study identifies new factors that act as barriers to parental consent and parents have for the first time been categorised into four decision‐making groups. Novel findings include that parents were 1.78 times more likely to consent to organ donation via the brain death pathway than via the circulatory death pathway. Little was previously known about the impact of donation type on consent in paediatric donation (Barber et al. 2006; Andrés et al. 2009). Qualitative findings revealed a range of interconnected factors that could positively or negatively influence parental consent decisions and wider family member influences. New factors not previously described in the paediatric literature include organ donation altering or prolonging timings in end‐of‐life care, the restricted time families have with their child after their heart stops for donation after circulatory death, negative parent and family experiences of the United Kingdom organ donation process, and integration and co‐ordination of healthcare teams in navigating organ donation.

Our findings also identified decision‐making factors that align with previous research on paediatric organ donation, thereby confirming their importance and suggesting that some factors are widespread irrespective of the local health and organ donation system. These factors include those that created barriers in parental consent to organ donation, such as surgery and the impact on a child's suffering, dignity and appearance (Yeşilbaş 2020; Bellali and Papadatou 2007; Hoover et al. 2014; Siminoff et al. 2001; Darlington et al. 2019), parallel stressors (Bellali and Papadatou 2007; Hoover et al. 2014; Rodrigue et al. 2008), family disagreement (Bellali and Papadatou 2007; Rodrigue et al. 2008), religious and cultural beliefs (Yeşilbaş 2020; Bellali and Papadatou 2007; Rodrigue et al. 2008; Mahat‐Shamir et al. 2019), non‐acceptance (Bellali and Papadatou 2007), mistrust (Bellali and Papadatou 2007), focus on specific organs that families do or do not want transplanted (Bellali and Papadatou 2007; Rodrigue et al. 2008; Mahat‐Shamir et al. 2019), and distress over organs being transplanted to an adult instead of another child (Rodrigue et al. 2008). Conversely, there were also factors that created facilitators to organ donation consent within parental decision‐making, including altruistic motivations (Darlington et al. 2019; Rodrigue et al. 2008; Luberda and Cleaver 2017), something positive coming of a tragic event (Hoover et al. 2014; Darlington et al. 2019), organ donation providing meaning to families (Siminoff et al. 2001), child's personality (Luberda and Cleaver 2017), trust in healthcare clinicians (Bellali and Papadatou 2007), and prior knowledge of organ donation and exposure to the benefits of organ transplantation (Yeşilbaş 2020; Bellali and Papadatou 2007; Hoover et al. 2014; Darlington et al. 2019; Rodrigue et al. 2008). Other factors were bidirectional, influencing family decision‐making either as barriers or facilitators depending on their nature. These include families' beliefs and behaviours about organ donation (Rodrigue et al. 2008; Luberda and Cleaver 2017), and clinician communication and quality of approach by the SNOD and healthcare professionals (Bellali and Papadatou 2007).

To date, only one other study by Godown et al. (2021) has conducted a quantitative analysis of paediatric organ donors similar to this study. Their analysis used regression models of routinely collected data from 11,829 potential paediatric donors in the United States (Godown et al. 2021). The quantitative findings from Godown et al. (2021) align with those of our study. However, a notable difference is that Godown et al. (2021) identified an association between the child's cause of death and the likelihood of parental consent for paediatric organ donation, with higher consent rates for deaths caused by trauma or suicide. While we initially replicated this finding in our unadjusted analysis, this association no longer held after adjusting for demographic variables. This suggests that the observed association between cause of death and consent outcome is confounded by other factors. Organ donation prolonging or altering timings was the most prominent factor influencing parent decision‐making, which is a novel factor that has not emerged as a barrier within previous research. Our findings indicate that discussions about organ donation often began only after timelines and plans for the withdrawal of life‐sustaining treatment had been established. Consequently, many parents and family members were reluctant to adjust their plans to accommodate the lengthy donation process. To address this, early notification practices for paediatric organ donation were implemented in April 2023 to promote organ donation being offered in parallel to other end‐of‐life care options. However, our data does not yet indicate whether this practice has influenced organ donation decision‐making by parents, and consent rates continued to decline in 2023/2024.

Tension and poor coordination between SNODs and paediatric intensive care unit (PICU) healthcare teams further impacted the timing and quality of organ donation approaches. One of the key challenges within paediatric organ donation is its rarity, and PICUs may only encounter a handful of potential donor cases annually (Brierley and Hasan 2012). SNODs may also lack experience and confidence in paediatrics, with previous research indicating nurses can feel anxious and unprepared when tasked with paediatric potential donors (Dopson and Long‐Sutehall 2019). In contrast, adult ICU is typically more focused on organ donation as an end‐of‐life option, and SNODs are more integrated within adult care teams; as a consequence, they spend less time in paediatric settings where organ donation is much less frequent. This can lead to weaker relationship‐building between SNODs and PICU, which may impact shared trust and the confidence of SNODs when working in paediatric environments. This may also partially explain why there are higher consent rates for children over the age of 16, who, from around age 16 years, are often cared for in adult ICU. These results suggest that current paediatric end‐of‐life pathways do not adequately integrate organ donation. A more proactive, coordinated approach involving all multi‐disciplinary team members involved in a child's care is needed to ensure families can fully consider organ donation as an end‐of‐life care option in conjunction with other options that may be available to them.

Some parents found the organ donation process overwhelming, information heavy, lengthy and distressing. Parents are approached about organ donation during the most challenging moments of their lives when they are already under immense strain. In this context, they are asked to make a critical and complex decision that can be both time‐consuming, challenging and emotionally taxing. Paediatric organ donation policies and practices should aim to minimise, where possible within the parameters of necessary procedural requirements the demands placed upon parents considering organ donation. Furthermore, research into the experiences of parents is needed to identify feasible changes that will reduce the burden some parents and families report. However, it is important to highlight that compared to other countries, the United Kingdom has a stringent and lengthy organ donation pathway, with certain aspects that cannot be modified unless the Human Tissue Act (2004) is amended (Price 2005; Ellis 2004). This act was introduced to regulate the use and storage of human tissues and organs after it emerged that organs had been removed at autopsy from children at Alder Hey Hospital in Liverpool without the knowledge and consent of parents (Bauchner and Vinci 2001). The updated Act has had a substantial impact on the length and content of the organ donation consent process in the United Kingdom as consent is required for every organ and tissue donated.

Our findings emphasise the importance of religious or cultural beliefs on parental decisions regarding organ donation, which was often linked with mistrust, dissatisfaction with care and disagreement about their child's prognosis. Clinicians could be hesitant to initiate or continue discussions about organ donation with parents and family members who have religious or cultural beliefs assuming likely refusal of consent, a bias that may be hindering optimal approach to end‐of‐life choices. Such assumptions by clinicians could also hinder equal access to end‐of‐life care choices. This aligns with a US study where potential donors from low‐consent groups, such as ethnic minorities, were less likely to be approached about organ donation for a child (Godown et al. 2021). Additionally, studies on adult organ donation show that Black families can experience poorer communication, receive less information, and report greater mistrust in the healthcare system (Siminoff et al. 2001, 2003, 2006). A contributing factor could be clinicians experience and confidence in navigating sensitive conversations around religion and organ donation. Notably, the 2019 strategy document for ‘UK Paediatric and Neonatal Deceased Donation’ does not address religion or ethnic minorities within its framework (Paediatric Subgroup of the National Organ Donation Committee 2019). Given the prominence of religion and ethnicity as factors influencing parental and family decision‐making, integrating this into future strategies could be an important next step.

Our analysis revealed that consent rates were lower among parents of younger children, and that these parents faced a higher proportion of certain factors that created barriers, including the impact of organ donation in prolonging or altering timings in end‐of‐life care, the need for surgery, and reduced time with their child after death. Reduced time with their child after death is a factor that creates a barrier associated with donation after circulatory death, which is more common among neonates and young children, explaining the higher prevalence observed. Additionally, it is understandable that parents may find the idea of surgery on a very young infant more distressing, making this factor more prominent in their decision‐making. Why a child's age influences parental decision‐making beyond the factors identified remains poorly understood. One theory derived from clinical experience suggests that SNODs often adopt a more cautious and guarded approach with parents of neonates or young infants as organ donation is less likely to be possible. This is typically due to poor utilisation of these small organs, primarily due to the technical aspects of retrieval and transplantation, a lack of approved organ preservation systems and suitable recipients.

Furthermore, current paediatric organ donation pathways and consent processes are largely modelled on adult pathways and are generally considered not to be optimally tailored to the unique circumstances of a child's death and the paediatric clinical context. For perinatal, neonatal, and paediatric palliative care guidelines in the United Kingdom, organ donation is poorly defined and only briefly mentioned (British Association of Perinatal Medicine 2024; Together for Short Lives 2015; Widdas et al. 2013; Larcher et al. 2015). Current care pathways also group donors and non‐donors together, which overlooks how organ donation greatly alters end‐of‐life care. There is also no age‐specific differentiation, and care pathways do not account for the distinct challenges and considerations involved in the decision‐making processes of parents for neonates, children, or young adults.

Our study demonstrates that the factors influencing parental decision‐making in paediatric organ donation are intricate and multifaceted, and four distinct decision‐making groups emerged in our analysis (Table 7). Mapping findings onto common theories used in this setting can help provide a deeper understanding of the overarching behavioural mechanisms at play. Here we consider three relevant theories:

1. ‘Psychological Stress and Coping’ theory suggests that individuals make decisions in stressful situations based on perceived demands and their available resources to cope (Biggs et al. 2017). Applied to organ donation, parents might decline donation if they perceive the additional stress as unmanageable and something that they cannot cope with. Our findings support this theory, as parents who were under enormous emotional strain or faced multiple parallel stressors often chose not to consent as they could not cope with the additional stress even if they supported the concept of organ donation.
2. Utilitarianism is an ethical theory that underpins organ donation and proposes that people will always make the best altruistic decision for themselves and their own situation rather than follow altruistic principles of helping others (Morris and Holt 2021; McLaughlin et al. 2025). Altruism by helping the transplant recipient was a prominent motivation for consent to organ donation and provided purpose and meaning to some parents and families who gave consent. However, for parents who chose not to consent, their altruistic intentions were refocused on protecting themselves, their child, and wider family from the stressors involved in and inability to cope with organ donation.
3. Decision‐making research on the role of reciprocity suggests that families with positive perceptions of the healthcare system and its staff are more likely to consent to organ donation due to increased trust and a sense of social responsibility (Van Doorn et al. 2014; Penner et al. 2005; López et al. 2018). Conversely, feelings of anger or frustration diminish this reciprocity, leading to non‐consent (Van Doorn et al. 2014; López et al. 2018). Our findings support this together with evidence in the wider literature. For example, a cross‐sectional study found that parents who consented to organ donation for their child reported higher satisfaction with healthcare services compared to non‐consenting parents (Rodrigue et al. 2008). The decline in public satisfaction with the NHS—from 60% in 2019 to just 24% in 2023—coincides with the decrease in paediatric organ donation consent rates, supporting the theory that negative perceptions of the health system may be influencing parents' willingness to consent (Jefferies et al. 2024).

Although direct paediatric‐specific comparison data are limited due to the lack of consistency in the way data are collected and reported, evidence of trends from countries with healthcare systems comparable to the United Kingdom indicate that the United Kingdom has lower organ donation consent rates for organ donation in both adult and paediatric settings (George et al. 2022). In the United States, the 2019 paediatric consent rate was 74%, substantially higher than in the United Kingdom, although wide variations were reported by state and other variables such as age (Godown et al. 2021). Furthermore, Spain, Australia and Canada, countries with similar healthcare systems to the United Kingdom, all currently report generally higher overall consent rates for organ donation. However, differences in data collection and reporting methods make direct comparisons difficult (George et al. 2022). In Australia, for example, paediatric organ donation applies to children aged 0 to 15 years (compared to 0 to 18 years in the United Kingdom), and between 2016 and 2019, although consent rates varied according to the approach used, the overall rate was higher than in the United Kingdom during a similar period (Cavazonni et al. 2023). In staff‐initiated conversations that involved a collaborative approach between the child's intensivist and the organ donation nurse, the consent rate was 60% (81 out of 136), compared with 25% (44 out of 178) for non‐collaborative approaches. Family‐initiated conversations achieved a consent rate of 81% (130 out of 160). Cross‐country comparisons can also be limited by cultural and healthcare system differences, such as the United States and Australia operating with a substantially different funding model to the United Kingdom. However, these contextual differences raise important questions about the factors and mechanisms influencing parental consent, and what lessons might be transferable to the United Kingdom for improving paediatric consent rates.

Existing theories and frameworks offer valuable insights, yet they do not entirely address the unique complexities of neonatal and paediatric organ donation, nor do they improve understanding about which targeted practices and policies could be used to move to a situation that supports a more bespoke approach and reduces barriers to consent.

### 5.1 Strength and Limitations

The mixed‐methods design combining quantitative and qualitative analyses provides a more comprehensive picture of the factors influencing parental consent to paediatric organ donation. Data were collected from the United Kingdom only, and some findings may not generalise to healthcare systems with different funding models in particular. There was a small number of children from minority ethnic groups, which required us to combine these children into a single non‐white category, preventing analysis across the different ethnic and cultural groups. Missing data were handled using listwise deletion. A higher proportion of missing ethnicity data was observed among potential donors where consent was not given, which may introduce bias into the analysis. Qualitative data were collected from the perspective of the SNODs and therefore do not include the perspectives of parents, families and other healthcare staff. This also introduces the risk of reporting bias regarding parents' experiences and the factors influencing decision‐making, as the perspective of SNODs will differ from those of parents. However, the SNOD perspective also offers unique advantages. We were able to capture observed clinical processes and interactions and analyse how these might affect parental decision‐making. SNOD practice regarding consent is heavily scrutinised, and as a result, SNODs will record as much information as possible providing a relatively detailed account of family decision‐making. Additionally, compared to previous studies, the SNOD perspective provided a potentially more objective view of parents' emotional and decision‐making processes. For example, compared to previous research, our findings have a stronger emphasis on the prevalence and impact of emotions such as anger, denial, or mistrust. SNOD notes were also recorded in near real time as soon as possible after the event, and so are less likely to be subject to recall bias than the down‐the‐line memories of grieving parents.

### 5.2 Recommendations for Further Research

Research is needed to explore the context and mechanisms that lead to different decision‐making outcomes. Qualitative research with parents and close family members is required to deepen our understanding of the organ donation decision‐making process, including their experiences with the healthcare system and organ donation process. In particular, insight into the decision‐making of parents who support organ donation yet encounter barriers (i.e., group 2 and 3) would help in developing targeted and tailored interventions. Future qualitative research should also examine specifically the decision‐making process of parents with younger children and those from ethnic minority or religious backgrounds.

The healthcare system and parent‐clinician communication are considered to play an important role in consent rates, as these factors can be modified through changes in clinical policy and practice (López et al. 2018). Future research should evaluate how various components of the healthcare systems (such as clinical pathways, consent processes, team integration and clinician communication) interact with parental decision‐making to identify intervention areas. Specifically, the early notification system for paediatric organ donation introduced in the United Kingdom in 2023 requires evaluation to determine its impact on consent rates.

Comparative research on paediatric organ donation practices and policies between the United Kingdom and countries achieving higher consent rates may also reveal modifiable factors to improve consent. Additionally, investigating regional variability in the United Kingdom paediatric consent rates could help identify regions, hospitals, or SNODs with particularly high consent rates and identify examples of innovative practice.

### 5.3 Implications for Policy and Practice

Ethically, interventions intended to increase organ donation consent must carefully balance supporting parents in their decision, without crossing into coercion. The four new classifications of parent decision‐making and outcomes (Table 7), in addition to new understandings about what influences parental decision‐making, can be used to inform and assist SNODs, clinical teams and palliative care specialists in how to tailor their approach to end‐of‐life care to achieve the best consent outcome for each child and their family. When more evidence is available, targeted interventions and more bespoke pathways could help to improve parent and family care and to create the conditions more likely to support consent to organ donation. These interventions are most likely to benefit parents in the decision‐making groups 2 and 3 (parents who are supportive of organ donation but faced critical barriers or serious concerns in their decision‐making).

Developing specific support strategies that acknowledge contextual, religious and procedural concerns may enhance consent rates and facilitate a more tailored parent‐ and family‐centred approach to paediatric organ donation and palliative care. For example, the development of communication strategies to address unique concerns across different cultures and religions. Timely and coordinated integration of organ donation into paediatric and neonatal end‐of‐life care that allows families to consider organ donation alongside other care options may be critical to improving consent rates. This study also highlights the need for a more coordinated and proactive partnership between PICU and the organ donation team to optimise organ donation integration and approach. The paediatric and neonatal end‐of‐life care pathways and organ donation processes could be further developed to better outline and integrate organ donation within clinical guidelines. Furthermore, improving the utilisation of organs from neonatal and very small donors needs to be addressed to improve the opportunities for parents of young children who want to choose organ donation as an end‐of‐life care pathway. The organ donation system generally could benefit from further evolution to alleviate parallel stressors, emotional distress and procedural concerns relating to organ donation (López et al. 2018; da Silva Knihs et al. 2020).

SNODs in the United Kingdom are highly trained healthcare professionals. However, evidence suggests that some SNODs lack confidence or experience in paediatric settings and are not currently trained in delivering psychological interventions to support families navigating the overwhelming stressors involved in the tragic death of their child (Dopson and Long‐Sutehall 2019; Scales 2024). Bespoke training could further enhance skilled clinician communication in building parental trust, shaping perceptions of organ donation, and fostering rational, altruistic decision‐making. Training should be informed by current research and could focus on psychological support skills, adaptation to the unique needs of different families, and navigating the challenging or sensitive situations frequently encountered in paediatric organ donation approaches.

## 6 Conclusion

Other countries such as the United States achieve better neonatal and paediatric consent rates, so there is potential opportunity to further improve policy, practice, and legislation in this area underpinned by a programme of research. Ethically, there is a fine line between support and coercion, and families have the right to choose what is best for their child and their family, which must be accounted for in the development of future policies and practices. Parents and close family members who have been approached about organ donation as an end‐of‐life care option for their child need to be involved in the evolution of future research, policies, and processes to ensure that they are child, parent and family friendly; that they take account of equity, diversity and inclusion; and that they are easily accessible to all professionals involved in the end‐of‐life care of children.

## Author Contributions

E.C., A.S., J.N., L.M., R.J.M. and R.H. made substantial contributions to conception and design, or acquisition of data, or analysis and interpretation of data. E.C., A.S., J.N., L.M., R.J.M. and R.H. were involved in drafting the manuscript or revising it critically for important intellectual content. E.C., A.S., J.N., L.M., R.J.M. and R.H. were given final approval of the version to be published. Each author has participated sufficiently in the work to take public responsibility for appropriate portions of the content. E.C., A.S., J.N., L.M., R.J.M. and R.H. agreed to be accountable for all aspects of the work in ensuring that questions related to the accuracy or integrity of any part of the work are appropriately investigated and resolved.

## Disclosure

Data statement: Any data utilised in this study has been lawfully acquired in accordance with The Nagoya Protocol on Access to Genetic Resources and the Fair and Equitable Sharing of Benefits Arising from Their Utilisation to the Convention on Biological Diversity.

Statistics statement: We confirm that we have checked to make sure that our submission conforms as applicable to the Journal's statistical guidelines. The statistics were checked prior to submission by Dr. Mayara Bianchim (email: m.silveirabianchim@bangor.ac.uk). We confirm that the methods used in the data analyses are suitably applied to their data within their study design and context, and the statistical findings have been implemented and interpreted correctly. We agree to take responsibility for ensuring that the choice of statistical approach is appropriate and is conducted and interpreted correctly as a condition to submit to the Journal.

## Ethics Statement

Ethical approval for this study was granted by the Schools of Medical & Health Sciences Ethics Committee, Bangor University (ref: 0187). Permission was also granted by NHSBT, and a data sharing agreement was signed by both parties.

## Consent

This study involved an analysis of routinely collected data, and so patient consent statements were needed.

## Conflicts of Interest

Prof Jane Noyes is an editor of the *Journal of Advanced Nursing*. However, she played no role in the processing of this manuscript. No other conflicts are declared.

## Supporting information


**Figure S1.** Timeline of important events in paediatric organ donation in the United Kingdom, and corresponding annual consent rates for paediatric organ donation.
**Table S1.** Good Reporting of a Mixed Methods Study (GAMMS) checklist.
**Table S2.** Initial and subsequent coding frameworks used in the qualitative analysis.
**Table S3.** Integration of quantitative results and qualitative findings.
**Table S4.** Factors that created barriers or facilitators to parental consent to organ donation for a child.
**Table S5.** Factors related to the healthcare system and clinician‐parent communication that created barriers or facilitators to parental consent to organ donation for a child.
**Table S6.** Frequency and proportion of cases where a factor was identified that created a barrier to, or facilitated, parental consent to organ donation, broken down by child age group.

## Data Availability

The data that support the findings of this study are not available to be shared due to privacy or ethical restrictions.
