# Supplementary material for: Understanding Why Parents Say Yes or No to Organ Donation When Their Child Dies: Mixed‐Methods Study
Source: J Adv Nurs. 2025 Jun 9;82(4):3617–38. doi: 10.1111/jan.17091 (PMC12994636; doi:10.1111/jan.17091)
Supplement: Supplementary file 1 — Figure S1. Timeline of important events in paediatric organ donation in the United Kingdom, and corresponding annual consent rates for paediatric organ donation. Table S1. Good Reporting of a Mixed Methods Study (GAMMS) checklist. Table S2. Initial and subsequent coding frameworks used in the qualitative analysis. Table S3. Integration of quantitative results and qualitative findings. Table S4. Factors that created barriers or facilitators to parental consent to organ donation for a child. Table S5. Factors related to the healthcare system and clinician‐parent communication that created barriers or facilitators to parental consent to organ donation for a child. Table S6. Frequency and proportion of cases where a factor was identified that created a barrier to, or facilitated, parental consent to organ donation, broken down by child age group. [file JAN-82-3617-s001.docx]

# Supplementary Information.

# Table of Contents

| *Page 2:* | Figure S1. Timeline of important events in paediatric organ donation in the UK, and corresponding annual consent rates for paediatric organ donation. |
| --- | --- |
| *Page 3:* | Table S1. Good Reporting of a Mixed Methods Study (GAMMS) checklist |
| *Page 4:* | Table S2. Initial and subsequent coding frameworks used in the qualitative analysis |
| *Page 8:* | Table S3. Integration of quantitative results and qualitative findings |
| *Page 9:* | Table S4. Factors that created barriers or facilitators to parental consent to organ donation for a child |
| *Page 18:* | Table S5. Factors related to the healthcare system and clinician-parent communication that created barriers or facilitators to parental consent to organ donation for a child. |
| *Page 21:* | Table S6. Frequency and proportion of cases where a factor was identified that created a barrier to, or facilitated, parental consent to organ donation, broken down by child age group. |

**Figure S1**. Timeline of important events in paediatric organ donation in the UK, and corresponding annual consent rates for paediatric organ donation.


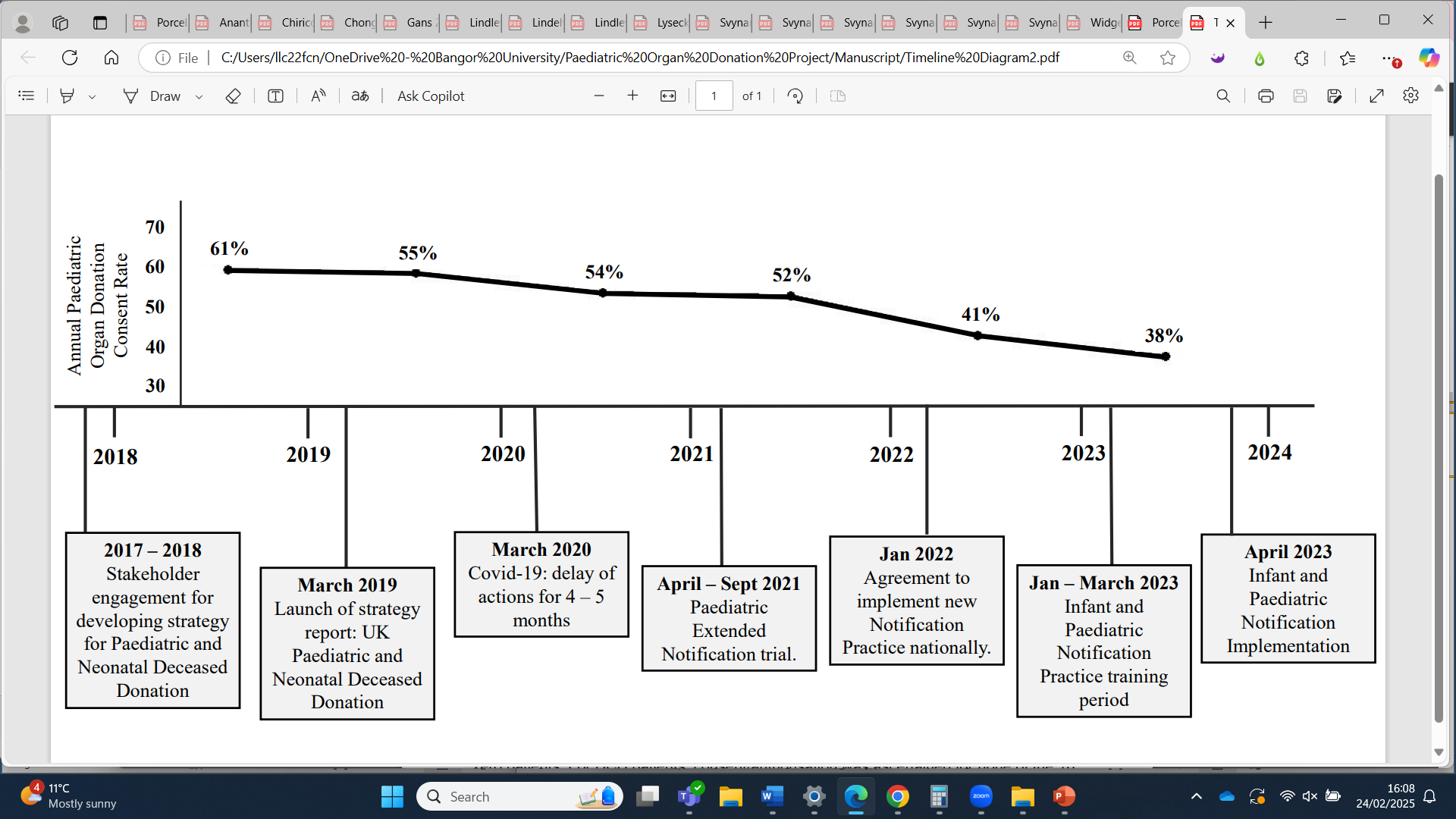


*Annual consent rates for paediatric organ donation are derived from the NHS Blood and Transplant annual report on donation and transplantation in paediatric patients. The annual period runs from April 1^st^ to March 31^st^ for each year.*

NHS Blood and Transplant. Annual report on donation and transplantation in paediatric patients [Internet]. Available from: https://www.odt.nhs.uk/statistics-and-reports/paediatric-activity-reports/

**Table S1**. Good Reporting of a Mixed Methods Study (GAMMS) checklist

| **Guideline** | **Section** |
| --- | --- |
| Describe the justification for using a mixed methods approach to the  research question | Methods in ‘Design’ section |
| Describe the design in terms of the purpose, priority and sequence of methods | Methods in ‘Data Integration’ section |
| Describe each method in terms of sampling, data collection and analysis | Methods from sections ‘Study Setting and Sampling to Quantitative analysis’ |
| Describe where integration has occurred, how it has occurred and who has participated in it | Methods in ‘Design and Data Integration’ section |
| Describe any limitation of one method associated with the present of the other method | Discussion in ‘Strength and Limitations’ section |
| Describe any insights gained from mixing or integrating methods | Results in ‘Integration of qualitative and quantitative findings’ section. |

O'Cathain A, Murphy E, Nicholl J. The quality of mixed methods studies in health services research. J Health Serv Res Policy. 2008;13: 92-98.

**Table S2.** Initial and subsequent coding frameworks used in the qualitative analysis.

| **Initial coding framework for high level coding.** | |
| --- | --- |
| **Code** | **Explanation** |
| Barrier or concern regarding consent to organ donation | Factors that created barriers to consent to organ donation, or concerns that families expressed about consenting to organ donation. |
| Factors that facilitated consent to organ donation or reason for wanting to donate. | Factors that created facilitators to consenting to organ donation or reasons families gave for choosing to consent. |
| Trust or mistrust | Examples of trust, or lack of trust, between families and healthcare professionals in the context of end-of-life care and organ donation decision-making. |
| Altruism | Altruistic motives in families' decisions to consent to organ donation for their children. |
| Grief, overwhelm, distress, or other emotional states. | Instances of families' emotional states in processing end-of-life care and deciding about organ donation for their child. |
| Family structures and dynamics | Family structures or dynamics and their potential impact on the parental decision-making process regarding organ donation for their child. For example, family conflicts, decision-making structures, or family circumstances. |
| Decision-Making Processes | Examples of the decision-making processes of families considering organ donation for their child. This is to examine how families weigh the decision of organ donation and how factors such as ethical, emotional, and personal aspects are considered. |
| Clinician communication and approach | Positive and negative examples of clinician communication and approach, particularly regarding organ donation, and the potential impact on the parental decision-making process. |
| Parallel stressors | Examples of the additional demands and stressors that families are faced with whilst also navigating end-of-life care and organ donation and the potential impact on the decision-making process. |
| Prior understanding of organ donation | The level of knowledge or awareness families have about organ donation prior to their approach. |
| Religion, culture, or personal spiritual beliefs | Identifying specific religious or cultural beliefs that impact families' attitudes towards organ donation, including interpretations of death, afterlife beliefs, and cultural practices. |
| Exhaustion | Examples of families being physically / emotionally exhausted. |
| End-of-life or palliative care preferences | Parents or child’s wishes for end-of-life or palliative care, and particularly how this relates to or impacts decision regarding organ donation for a child. |

**Subsequent coding framework for more nuanced coding**

| **Code** | | **Explanation** |
| --- | --- | --- |
| Barrier or concern regarding consent to organ donation | | Factors and contexts that created barriers to consent to organ donation, or concerns that families expressed about consenting to organ donation. |
|  | Organ donation prolonging or altering timings for when child dies. |  |
|  | Surgery, further medical operations, and the impact of surgery on the child’s body |  |
|  | Concerns about child’s suffering |  |
|  | Fate of the organs |  |
|  | Parental disagreement about consenting to organ donation |  |
|  | Wanting to stay with child after death |  |
|  | Translator needed |  |
|  | Misunderstanding or confusion. |  |
|  | Keeping the child whole |  |
| Factor that facilitated consent to organ donation or reason for wanting to donate. | | Factors that created facilitators to consenting to organ donation or reasons families gave for choosing to consent. |
|  | Altruism | Altruistic motives in families' decisions to consent to organ donation for their children. |
|  | Something positive |  |
|  | Keeping the child whole |  |
|  | Child previous expressed wanting to donate |  |
|  | Parents felt that the child would have wanted organ donation |  |
|  | Child’s personality |  |
|  | Previous or potential beneficiary of organ donation. |  |
|  | Organ donation as a source of pride or legacy. |  |
| Emotional factors | | Instances of families' emotional states in processing end-of-life care and deciding about organ donation for their child. For example, grief, overwhelm, distress, or other emotional states. |
|  | Anger or frustration |  |
|  | Unacceptance |  |
|  | Overwhelm and distress |  |
|  | Mistrust | Mistrust of clinicians or the healthcare service. |
| Family structures and dynamics | | Family structures or dynamics and their potential impact on the parental decision-making process regarding organ donation for their child. For example, family conflicts, decision-making structures, or family circumstances. |
|  | Parental disagreement about whether to consent to organ donation. |  |
|  | Complex or challenging family circumstances |  |
| Decision-Making Processes | | Examples of the decision-making processes of families considering organ donation for their child. This is to examine how families weigh the decision of organ donation and how factors such as ethical, emotional, and personal aspects are considered. |
| Clinician communication and approach | | Positive and negative examples of clinician communication and approach, particularly regarding organ donation, and the potential impact on the parental decision-making process. |
|  | Examples where clinician communication or approach facilitated parental consent for organ donation. |  |
|  | Examples where clinician communication or approach created barriers to parental consent for organ donation. |  |
| Parallel stressors | | Examples of the additional demands and stressors that families are faced with whilst also navigating end-of-life care and organ donation and the potential impact on the decision-making process. |
| Prior understanding of organ donation | | The level of knowledge or awareness families have about organ donation prior to their approach. |
|  | Parents or family members raised organ donation before clinicians approached formally. |  |
|  | Parents or family expressed that they had already thought about organ donation. |  |
| Religion, culture, or personal spiritual beliefs | | Identifying specific religious or cultural beliefs that impact families' attitudes towards organ donation, including interpretations of death, afterlife beliefs, and cultural practices. |
| Exhaustion | | Examples of parents being physically or emotionally exhausted. |
| End-of-life or palliative care preferences | | Parents or child’s wishes for end-of-life or palliative care, and particularly how this relates to or impacts decision-making regarding organ donation for a child. |

*This table outlines the coding frameworks used to analyse factors influencing parental decision-making regarding organ donation for their child. The initial framework, informed by stakeholder engagement and previous research, was applied during the first analysis of the qualitative data. The subsequent framework was developed iteratively through deeper familiarisation with the data to further categorise findings into sub-factors and to better understand their associations and context.*

**Table S3.** Integration of quantitative results and qualitative findings

| **Factor** | **Quantitative analysis** | **Qualitative analysis** | **Implications** |
| --- | --- | --- | --- |
| Donation by circulatory death | Donation by brainstem death is associated with higher odds of consent compared to donation by circulatory death (adjusted odds ratio = 1.78, p = 0.003). | Families experienced logistical challenges with donation after circulatory death which impacted the parents experience of end-of-life care of their child.   - With donation by circulatory death families are restricted to a 5-minute window after their child’s heart stops before the child is taken to the operation theatre, which many parents struggled to accept. - For donation after circulatory death, parents are approached about organ donation whilst the child is still alive, which could be distressing for parents. | The timing and procedural differences associated with donation by circulatory death likely have a negative impact on the likelihood of parental consent, resulting in the observed lower odds of consent for donation by circulatory death. |
| Religious/cultural beliefs and ethnicity | Parents from non‑white ethnic backgrounds are significantly less likely to consent (adjusted odds ratio = 5.03, p < 0.001). | Religious and cultural beliefs, including wider family/community influences, were identified as common factors in decisions not to consent. | It is plausible that ethnicity and religious/cultural factors are associated or are related in their influence on parental consent outcomes. This is based on the theory that minority ethnic groups are more likely to be religious. |
| Age of the child | The odds of consent increase by 6% with each additional year of the child’s age (adjusted odds ratio = 1.06 per year, p < 0.001), indicating lower consent rates for younger children. | Barriers such as altered end‑of‑life care timings, aversion to surgery, and limited time with the child after death were more common among parents of younger children. For example, 31% of parents with a child under 1 year reported such barriers, compared to 16% in older age groups. | Parents with younger children experience a higher incidence of certain barriers, which may contribute to the lower consent rates observed among these families. |

*This table integrates quantitative results with qualitative findings on factors influencing parental consent for organ donation for their child. For each factor, quantitative results and qualitative findings related to similar phenomena of interest are presented, and the insights and implications are presenting in the final column.*

**Table S4.** Factors that created barriers or facilitators to parental consent to organ donation for a child.

| **Factors that created barriers to parental consent for organ donation** | | |
| --- | --- | --- |
| **Theme** | **Findings** | **Quote** |
| **Organ Donation Prolonging or Altering Timings** | Concerns about the disruption or prolongation of timelines was prevalent barrier. A plan for the withdrawal of life support (WLST) had frequently been made prior to organ donation being raised. Many parents wanted withdrawal of life supporting treatment to occur as soon as possible or as previously planned. The emotional strain of delaying withdrawal of life supporting treatment was often cited, as parents found the extension of the process distressing. Additionally, logistical challenges further complicated parents willingness to delay withdrawal of life supporting treatment. | “Plans for the withdrawal of life supporting treatment had already been made prior to offering organ donation. Parents are expecting the withdrawal of life supporting treatment today.”  “Initially consented and later declined. Family felt the length of time for the donation process was too long.”  “Mum and Dad stated that they needed closure today.”  “The parents did not want to prolong the situation any longer and wanted withdrawal of treatment.” |
| **Religion and Cultural Beliefs** | Religious and cultural beliefs often led to non-consent. Many parents believed organ donation conflicted with their religious views and had concerns about the opinions of faith leaders and their community. Religion was also linked to mistrust and non-acceptance of prognosis. In some cases, clinical teams appeared hesitant to raise or further discuss donation when religion was raised as a barrier. Religious parents who consented often did so with the integration of religious rituals and practices, though the specific reasons for consent were not always clear in the notes. | “The family wanted him to remain whole, as they are unaware what happens in the afterlife.”  “Strong no from Mum. Upon probing further, we found that this was because the parents religion/culture did not allow organ donation. I discussed the comfort other Islamic families have found knowing another child's life could be saved. End-of-life care was discussed further with plan for withdrawal of life-supporting treatment…. [later on] We received a call back from the unit, and the parents have changed their mind and are now supportive of organ donation. Islam faith important.”  “The family did not want the body touching after death and they required the child to remain whole to go to Allah. They specified they did not want a postmortem and would be exploring this further with the coroner or police should it be necessary. Doctor felt it was an appropriate time to bring in organ donation and sensitively explore. The male members of the family strongly declined expressing it was against their religious beliefs.”  “Mum was very distressed and expressed that God was in charge and that he will save child along with the belief that he still had life in him. I acknowledged this but again reiterated that the neurological determinant of death test would confirm death. Mum continued to say God would save her son.” |
| **Surgery or the impact of surgery on the child’s body or appearance** | Concerns about surgery and its impact on the child’s body were frequently raised. The thought of surgery evoked emotional reactions with parents often using terms such as "butchered" or "cut up". Parents often had strong aversion to changes to the child’s body or the thought of the child undergoing more procedures.  Discussions with healthcare professionals emphasizing the dignity of the surgery and the ability to conceal incisions helped some parents to reframe their perception of surgery. | “Mum stated that she knew this would be a good thing to do, but the thought of child being cut up made her feel sick.”  “Approached for organ donation with an immediate negative answer from both parents stating that they did not want her to be cut up. I reassured them that we would take good care of her, and that the operation was like any other operation, performed by specialist surgeons. It would involve a clean single incision, which will be stitched and covered with a dressing that she would look the same once dressed. Family aware of the benefits for recipients but they apologised and said that although they might donate themselves, they could not do it with child.”  “Dad's last issue was surrounding the surgery, and he used the term "butchered" we quickly dispelled this view and explained that the operation conducted was the same as any other procedure and that it was undertaken by specialist transplant surgeons. Dad accepted this and understood.” |
| **Concerns about Suffering** | Parents often felt their child had suffered enough and wanted to let them go in peace. Parents worried about causing additional suffering or discomfort to the child. | “Mother expressed that she thinks he has suffered enough, and she does not want him to be in any pain. The Consultant in Charge told the parents that the child is not in any pain, and he doesn't think he's felt any pain for a while. Mum understands this, and profusely apologised for not wanting him to donate his organs.”  “Mum and dad positively regard organ donation, however respectively declined organ donation. The family have already discussed it and they feel child has been through enough.”  “Dad has said that he cannot bear to see his son go on like this any longer…. He told the Consultant in Charge that he knew his baby could go on to save others and help other children and that he knew that was the right thing to do, but that no one has any idea what he has been through recently and he simply cannot tolerate anything else happening to him.” |
| **Non-acceptance and acute distress** | Non-acceptance was often associated with parents being distraught, in denial, and unable to process the situation. Non-acceptance made it challenging for parents to process information and engage in discussions about end-of-life care and organ donation. Non-acceptance was frequently associated with acute distress.  Given the trauma and shock of a child’s death, many parents struggled to cope, feeling shell-shocked and distraught. Parents experiencing this level of distress were often withdrawn, avoiding eye contact, and finding it difficult to engage in discussions. Many of these parents expressed the need for more time and space.  Some parents gave consent after they had more time to process and accept their child's condition. For those who consented, having time and space helped them process and come to terms with the situation. However, for parents who did not consent, discussions about organ donation only heightened their distress, leading to further disengagement. | “Mum, dad and teenage sister distraught, unable to process anything, and in denial about the confirmatory nature of the tests.”  “Acknowledgement from all except mum was given. Mum remained silent and expressionless. Family was processing information when Mum began screaming and rocking in the seat, visibly distressed… Mum was not accepting of the information and remained very distressed. Later on Mum was much calmer and expressed that child was gone and the waste of his life. At this point I approached for organ donation. Mum was quite receptive to this and as I discussed further the benefits of organ donation and how he could help others live on. She was keen for organ donation to be pursued.”  “Mum asking if more time will help, that people wake from comas, and he has only been here a short time…. The conversations were challenging for the Consultant in Charge.”  “Patient's mother is unsure about consenting as the patient's father is not accepting of the prognosis.” |
| **Anger** | Anger manifested in several ways amongst the parents who were approached about organ donation.   - Anger upon being approached for organ donation, with parents perceiving it as an intrusion or insensitive. - Anger with the quality of care or feelings of being let down by the service. This included previous negative experiences, feeling misled or misinformed, and perceived failure of services in treating their child. - Anger related to disagreement with care or prognosis, with some parents feeling that discussing organ donation implied giving up on their child. - Anger about the inability to protect their child and the difficult situation they found themselves in.   Among parents who consented, anger was more often directed at the situation itself, such as the rapid deterioration of their child, rather than at the service. | “Difficult complex medical case. Mum wishing to sue trust and is extremely unhappy about care. Mum stated she did not wish to speak to organ donation team”.  “Family angry at being offered donation, comparison to vultures. Difficulty for SNOD to speak personally to the parents.”  “I spoke to mum and despite her being angry about being in the situation she is in, she wants the child to help others in any way possible. She is wishing she had not let him be transferred to [hospital name] as she felt she was too unstable and that due to her instability she has not been able to receive the treatment she was transferred for. We tried to comfort her by saying that she had given the child the best chance possible.”  “Dad is very upset by the care given by the mental health facility. He felt the hospital let them down.”  “Family wished for coroner to investigate, felt the system had failed them and that their child’s death was preventable. This continued throughout all end-of-life care decisions.” |
| **Mistrust** | Parents expressed doubts regarding neurological test accuracy, questioned healthcare motivations, and sought second opinions. Mistrust was sometimes linked to perceptions that staff were giving up too soon, previous negative healthcare experiences, and religious beliefs. When parents became untrusting, healthcare staff frequently slowed down conversations or adopted a consultant-only approach. | “Family believes that patient's treatment may have been limited to facilitate organ donation.”  “Parents asked if organ donation was money driven.”  “Some distrust from parents around specific tests. This appeared to come from outside advice from family members.”  “I made the family aware that organ donation is a process which can take upwards of 24 hours, but this isn’t a decision that needed to be made there and then... Mum questioned if something was being kept from them as a family, if the team had a specific timeframe in mind, and if we felt his heart wouldn't last.”  “Family have informed consultant in charge that they have been watching child move and open her eyes… Family have also enquired about a second opinion but don’t want the doctors here to organise it. They have also reported that they will get in touch with legal advisors in relation to care to stop the unit potentially testing.” |
| **Fate of the Organs** | Parents expressed various anxieties regarding the fate of the organs, including:   - The risk of passing on underlying conditions to recipients, and thus feeling responsible for another child’s health. - The viability of the organs and the potential for unsuccessful transplantation. - Concerns about the anonymity of transplant recipients, including whether or not they wanted correspondence with them. - Refusal of consent as parents wanted the organs transplant to be to another child, which could not be guaranteed under the NHS priority system. | “Information from oncologist suggested some risk of transmission and family felt ‘they could never forgive themselves if this happened' … Family declined mid process on risk of transmission.”  “Mum had lots of concerns and the main concern being the possibility of organs been retrieved and not transplanted. I reiterated that she was young and healthy until this admission and that the chances of kidneys and liver being successfully transplanted were extremely high. However, I could not guarantee this with 100% certainty. Mum then apologised and said that she was going to say no as she needed 100% certainty”.  “Parents had some concerns in regard to the unconditional nature of organ donation. They felt uneasy about the anonymity of the recipients, the fact that the organs could go onto an adult and the uncertainty of receiving or not correspondence from the recipients.” |
| **Parental disagreement about organ donation** | Family disagreement often impeded parents ability to reach a consensus. For those who consented, family members were sometimes at different stages of acceptance and grief, requiring time to reach a point where they could make a decision or support consent. | “They stated they would have liked to be able to say yes but Mum particularly struggling. Dad ultimately agreed to support Mums decision.”  “Understand benefits and that it was a good thing to do. Changed mind several times. Appears split in family and some persuasion from other family members.”  “Parents disagreed and one wanted to overrule the other - not able to seek consensus.” |
| **Complex or Challenging Family Circumstances** | Complex family dynamics, such as conflict, parental separation, social service involvement, and substance use, hindered discussions about organ donation. In some cases, only male family members were allowed to make the decision. | “…there is a very large extended family, who are not accepting, and hoping for miracles, and who are influencing the parents.”  “He advised that the family have a lot of external factors and that Mum is being blamed for what has happened by Dad's parents.”  “Mum said that only herself, dad and the children knew about organ donation and to please not to mention it to the rest of the wider family as they had different views about it”  “Family dynamics are extremely complicated. Other family members are in adult intensive care unit from the same accident. Siblings have yet to be informed. Parents also require separate conversations.”  “Child is looked after by local authorities. Mum has a mental illness”. |
| **Staying with child after death** | For Donation following Circulatory Death, the time parents can spend with their child after the heart stops is limited to 5 minutes before the child is taken to the operating theatre. Parents refused consent to organ donation as they were concerned about the limited time they would have with their child and the distress this would cause. | “Mum can’t say goodbye to him in 5 minutes”.  “We then discussed the 5-minute period following asystole. Mum became very upset and said she wanted to be with her and hold her after she had died and that she couldn't cope with the thought that she would be taken away so quickly. Mum and Dad agreed that they would not be able to support donation and wanted to be able to spend time with her and cuddle her without all the tubes after she died.”  “Parents unanimously in agreement that the logistics of time associated with Donation after Circulatory Death wouldn't be in keeping with what they want end-of-life care to look like. The idea of 5 mins 'hands-off' before child transferring to theatre would be further unnecessary distress and they've already been through a lot.” |
| **Translator needed** | When interpreters were used, there were recorded concerns about the accuracy of information being relayed. In all instances where parents consented, parents members were translating for other relatives, suggesting that some members of the parents understood English. Additionally, there may have been other cultural and religious barriers that contributed to parents decisions not to consent, beyond the issues related to translation. | “An interpreter used, and there are some concerns about relaying of information correctly”. |
| **Misunderstanding or confusion** | Confusion primarily centred on understanding neurological determination of death (NDT), as well as the two pathways for organ donation (Donation following brainstem death and donation following circulatory death). | “Family understanding and expectations regarding the timings of brainstem death and withdrawal of life-sustaining treatment were not clear”.  “Some confusion around organ donation pathways”. |
| **Emotional and physical exhaustion** | Most parents experiencing emotional or physical exhaustion still consented to organ donation. While exhaustion may have delayed discussions or made the process more challenging, it did not seem to prevent parents from ultimately consenting. | “Patient's father stated that they all feel exhausted”. |
| **Focus on specific organs** | For parents who consented to organ donation, 26 expressed specific preferences regarding which organs were or were not donated. Most parents did not want certain organs, such as the corneas, heart, bowel, or tissue, to be donated. This reluctance was often due to concerns about taking organs that would be outwardly visible or cause a change in the abdominal shape of their child. Conversely, some parents were keen for specific organs, like the heart, to be donated or were disappointed when they could not be. | “Mum response was positive and said, "only don’t take his eyes"”.  “Organ donation is something both parents want to support, but they do not want to consent to heart. They talked about the symbolism of the heart.”  “Declined tissue and bowel as too much and did not want anything taken that was 'outwardly visual’”. |
| **Parallel stressors** | The SNOD notes highlighted a range of additional stressors or concerns that parents had to manage alongside the decision-making process. These stressors included media or social media attention, support for siblings, the presence of other seriously ill or injured family members, complex family dynamics, pregnancies, travel to the hospital, family conflicts, mental health issues, and criminal proceedings or police involvement. These parallel stressors appeared to intensify an already overwhelming situation, making it more challenging for parents to consider, consent to and accommodate organ donation. To help parents manage these difficult dynamics, certain measures were noted as facilitators. For instance, accommodations to help parents navigate complex family dynamics, childcare support, psychological care, or accommodations for families with mental or physical health conditions. | “…dad led the conversation explaining that currently they have no address with the house being burned down.”  “Safeguarding and police involvement.”  “Family distress at postmortem and geographical distance of this location from their home.”  “Unexpected death. Dad working away and had to fly home. Mum is heavily pregnant.”  “Dad visiting from prison. Documented approach sped up due to prison time constraints.”  “Parents supporting two other siblings. Worried around what to tell them and when.” |
| **Factors that Create Facilitators to Organ Donation** | | |
| **Altruism** | The desire to help other families and children was mostly expressed by parents and, in some cases, by the child’s siblings. Some parents were motivated by the hope that their decision to donate could prevent other families from experiencing the same pain they were going through. Parents sometimes expressed a desire for their child's life not to be in vain and to help others in some way. | “He is gone, and we want to do whatever we can to help others.”  “Although devastated the parents are adamant that they would like to help others. In fact mum asked that we 'do everything overnight to make it possible for her to donate her organs'. Mum has said she will get comfort from this”.  “Dad simply stated ‘they would be saving another family from going through this experience’. This seemed to cement Mum's thoughts that this was the right thing and what he had wanted.”  “Dad had some concerns about timeframes. Mum and sister felt helping others was most important.” |
| **Something positive** | When consenting to organ donation, having something positive come from a very difficult and tragic event was frequently a reason/facilitator to consenting to donation. Parents cited that they didn’t want their child’s life to be a waste, they wanted something good to come from their child’s life. | “Parents cried with relief over the positive option.”  “Have made them aware of potential timings and they agree that for something good to come out of this they can wait for organ donation.” |
| **Child wanted or would have wanted organ donation.** | In deciding whether to consent to organ donation, parents often considered what their child would have chosen, or their personality. Many parents felt that their child would have wanted to donate, using memories and aspects of their child's personality to guide their decision. The child’s personality was often expressed in parents decision to donate, citing that they were kind, caring, loving, or giving. It was often recorded that organ donation was seen as a way to honour the child's legacy and for the parents to feel pride.  In some instances, the child had explicitly expressed or registered their wish to donate their organs. This was frequently for altruistic reasons. In all cases, parents honoured their child decision to donate. | “Brother and others in room convinced this is what child would want as "has heart of gold". All in agreement that he would want something positive to come out of this.”  “Family raised organ donation during conversations as they had watch a programme together and their child had expressed a wish to donate. Keen to follow child's wishes.”  “Raised by Mum as they had discussed organ donation when child applied for driving licence. Child wished to help others. Family fully supportive of this wish” |
| **Prior knowledge or discussion of organ donation** | 78 parents or family members were recorded as having raised organ donation prior to being approached. This indicates prior knowledge or discussion of organ donation. The vast majority of these parents consented to organ donation. | “Parents had raised donation in a positive way and wished for more information regarding the process.”  “All family voiced that they would find comfort in organ donation, and they are on the organ donation register themselves.” |
| **Child or family member previous or potential beneficiary of organ donation** | There were families where a child or family member was a previous or potential beneficiary of organ donation. Many of these parents expressed that they would have accepted an organ transplant for their child, and so they felt it was right to also be willing to help and choose to donate organs for other families. | “Their child would have potentially needed an organ transplant. The parents would have accepted a transplant, so they felt it is only right to offer to help others too.”  “Child had two heart transplants and parents felt it was the right thing to do.” |

Quotes have been edited for grammar, readability, and to protect anonymity of the child and their parents/family. SNODs = Specialist Nurse in Organ Donation. Consultant in Charge is the term used for a senior doctor in the United Kingdom that has fully completed medical training.

**Table S5.** Factors related to the healthcare system and clinician-parent communication that created barriers or facilitators to parental consent to organ donation for a child.

| **Theme** | **Factors that created barriers to consent** | **Factors that created facilitators to consent.** | **Quotes** |
| --- | --- | --- | --- |
| **Parents experiences of the organ donation consent process** | Some parents reported feeling overwhelmed or upset by the extensive information provided and the numerous questions asked during organ donation discussions and the consent process. | Some parents who felt overwhelmed or upset, requested minimal details and engagement, specifically about surgery or specific organ. Accommodating this appeared to help parents remain supportive of their decision to consent. | “They had fatigue with questions and stated no to tissues. They did not wish for any information regarding retrieval operation.” |
| **Integration between main healthcare team and SNODs** | Tensions between the main healthcare team and Specialist Nurse in Organ Donation (SNOD) obstructed the integration of organ donation into end-of-life care. This included reports of:  In several cases there seemed to be a disconnect between SNODs and the main healthcare team. This appeared to be most apparent in clinical plans not being followed in collaboration with the SNODs. |  | “Consultant in charge declined to have SNOD present for first conversation due to 'longstanding conflict between paediatric intensive care unit and SNODs'. Donation was raised days later by SNOD.”  “Paediatric intensive care unit 'territorial' over family. SNOD asked by the paediatric intensive care unit to leave the conversation”. |
| **Communication between clinical staff and parents** | Some parents were reported to have experienced insensitive communication from the clinical team. For example, in one case, “family stated they felt rushed to end her life” and another family were recorded as saying, “one of the consultants were cold and clinical”. | There were several examples of SNODs challenging assumptions held by other members of staff. | “At present child is showing some positive signs (e.g. coughs, spontaneous breaths) which Dad would like this investigating further…. [later] Dad was understanding of the change of treatment plan and was grateful for our openness and honesty. Dad stated that he takes comfort knowing that we are investigating fully.” |
| **Assumptions by clinical staff about whether parents would consent to organ donation.** | In a few instances, clinicians assumed that parents would not want to consider organ donation, and so it would not be appropriate to ask them, thus preventing these parents from having a choice about consenting to organ donation as an end-of-life care option for their child. | There were several examples of SNODs challenging assumptions held by other members of staff. | “Bedside nurse mentioned that she believes family would decline organ donation due to their religion (Islam). I replied cautiously and argued that this doesn't mean they would be against organ donation. However if the nursing staff and medical staff could keep organ donation in mind for future planning it would be really positive for end-of-life planning, enabling his family to have a choice. Bedside nurse agreed positively.” |
| **Familiarity of staff** | Several parents found the involvement of numerous different staff members to be a source of added stress. | For parents who consented, familiar staff was a source of comfort in navigating the organ donation process. | “Familiarity of staff was important to the parents. Father expressed no one new to enter room”.  “Family found comfort in a SNOD remaining with their child in theatre”. |
| **Emotional reactions of clinicians** | In two cases, clinicians expressed feeling emotionally conflicted about organ donation for a child under their care. This emotional conflict could have had a negative impact on the families' perception of organ donation. |  | “Bedside nurse felt she was keeping the child going for organ donation. Consultant in Charge was also emotional as he had brought the child in by transfer”. |
| **End of life support for families.** |  | End of life preferences and wishes were very important to parents and were a great source of comfort in saying goodbye to their child. SNODs were proactive in offering options and seeking to understand end of life preferences and wishes.  SNODs explained the process and options for organ donation, helping parents make informed choices about how this pathway would impact end-of-life processes. Where possible, SNODs worked to align organ donation with the families wishes. In some cases families were given the option of donation following circulatory death or donation following brain death which helped some parents to overcome barriers to organ donation. | “Mum expressed desire to be present when the child’s heart stops, therefore donation after circulatory death pathway was offered. Donation after brainstem death was discussed at some point, and the parents were open to this as it would enable heart donation. Ultimately parents decided to proceed with donation after circulatory death pathway so they can be with the child when their heart stops.” |

Quotes have been edited for grammar, readability, and to protect anonymity of the child and their parents/family. SNODs = Specialist Nurse in Organ Donation. Consultant in Charge is the term used for a senior doctor that has fully completed medical training in the United Kingdom.

**Table S6.** Frequency and proportion of cases where a factor was identified that created a barrier to, or facilitated, parental consent to organ donation, broken down by child age group.

| **Factors that created barriers to parental consent to organ donation for a child** | | | | | | | | | | | | | | | | | | | | | |
| --- | --- | --- | --- | --- | --- | --- | --- | --- | --- | --- | --- | --- | --- | --- | --- | --- | --- | --- | --- | --- | --- |
|  | **Frequency and proportion of cases where factors that created barriers to parental consent for organ donation were identified** | | | | | | | | | | | | | | | | | | | | |
|  | **Under 1 years of age** | | | | | **1 – 10 years of age** | | | | | | **11 – 15 years of age** | | | | | | **16 – 17 years of age** | | | |
|  | **Parents who did not give consent to organ donation (n = 71)** | **Parents who did give consent to organ donation (n = 31)** | | | **Total (n = 102)** | **Parents who did not give consent to organ donation (n = 109)** | | | **Parents who did give consent to organ donation (n = 89)** | | **Total (n = 198)** | **Parents who did not give consent to organ donation (n = 77)** | | | **Parents who did give consent to organ donation (n = 83)** | **Total (n = 160)** | | **Parents who did not give consent to organ donation (n = 45)** | **Parents who did give consent to organ donation (n = 89)** | **Total (n = 134)** | |
| Organ donation prolonging or altering timings for when child dies. | 22 (31%) | 2 (6%) | | | 24 (24%) | 22 (20%) | | | 15 (17%) | | 37 (19%) | 14 (18%) | | | 15 (18%) | 29 (18%) | | 7 (16%) | 3 (3%) | 10 (7%) | |
| Religion and cultural beliefs | 10 (14%) | 2 (6%) | | | 12 (12%) | 28 (26%) | | | 9 (10%) | | 37 (19%) | 21 (27%) | | | 3 (4%) | 24 (15%) | | 11 (24%) | 10 (11%) | 21 (16%) | |
| Surgery, further medical operations, and the impact of surgery on the child’s body | 12 (17%) | 2 (6%) | | | 14 (14%) | 18 (17%) | | | 8 (9%) | | 26 (13%) | 7 (9%) | | | 10 (12%) | 17 (11%) | | 5 (11%) | 5 (6%) | 10 (7%) | |
| Non-acceptance and acute distress | 7 (10%) | 3 (10%) | | | 10 (10%) | 16 (15%) | | | 17 (19%) | | 33 (17%) | 9 (12%) | | | 7 (8%) | 16 (10%) | | 3 (7%) | 10 (11%) | 13 (10%) | |
| Concerns about child’s suffering | 13 (18%) | 4 (14%) | | | 17 (17%) | 17 (16%) | | | 7 (8%) | | 24 (12%) | 11 (14%) | | | 5 (6%) | 16 (10%) | | 7 (16%) | 1 (1%) | 8 (6%) | |
| Fate of the organs | 5 (7%) | 3 (10%) | | | 8 (8%) | 4 (4%) | | | 7 (8%) | | 11 (6%) | 3 (4%) | | | 4 (5%) | 7 (4%) | | 2 (4%) | 5 (6%) | 7 (5%) | |
| Anger | 1 (1%) | 2 (6%) | | | 3 (3%) | 9 (8%) | | | 2 (2%) | | 11 (6%) | 7 (9%) | | | 4 (5%) | 11 (7%) | | 3 (7%) | 4 (4%) | 7 (5%) | |
| Physical or Emotional Exhaustion | 2 (3%) | 0 (0%) | | | 2 (2%) | 0 (0%) | | | 7 (8%) | | 7 (4%) | 4 (5%) | | | 6 (7%) | 10 (6%) | | 2 (4%) | 5 (6%) | 7 (5%) | |
| Parental disagreement about consenting to organ donation | 0 (0%) | 2 (6%) | | | 2 (2%) | 9 (8%) | | | 2 (2%) | | 11 (6%) | 5 (6%) | | | 4 (5%) | 9 (6%) | | 2 (4%) | 1 (1%) | 3 (2%) | |
| Wanting to stay with child after death | 9 (13%) | 1 (3%) | | | 10 (10%) | 7 (6%) | | | 3 (3%) | | 10 (5%) | 4 (5%) | | | 4 (5%) | 8 (5%) | | 1 (2%) | 0 (0%) | 1 (1%) | |
| Mistrust | 1 (1%) | 1 (3%) | | | 2 (2%) | 9 (8%) | | | 1 (1%) | | 10 (5%) | 4 (5%) | | | 2 (2%) | 6 (4%) | | 6 (13%) | 1 (1%) | 7 (5%) | |
| Complex or challenging family situations | 0 (0%) | 0 (0%) | | | 0 (0%) | 5 (5%) | | | 4 (4%) | | 9 (5%) | 6 (8%) | | | 4 (5%) | 10 (6%) | | 1 (2%) | 1 (1%) | 2 (1%) | |
| Translator needed | 3 (3%) | 0 (0%) | | | 3 (3%) | 5 (5%) | | | 1 (1%) | | 6 (3%) | 1 (1%) | | | 2 (2%) | 3 (2%) | | 0 (0%) | 1 (1%) | 1 (1%) | |
| Keeping the child whole | 4 (4%) | 1 (3%) | | | 5 (5%) | 3 (3%) | | | 0 (0%) | | 3 (2%) | 3 (4%) | | | 1 (1%) | 4 (3%) | | 0 (0%) | 0 (0%) | 0 (0%) | |
| Misunderstanding or confusion | 0 (0%) | 0 (0%) | | | 0 (0%) | 4 (4%) | | | 2 (2%) | | 6 (3%) | 0 (0%) | | | 0 (0%) | 0 (0%) | | 0 (0%) | 0 (0%) | 0 (0%) | |
| **Factors that created barriers to parental consent to organ donation for a child** | | | | | | | | | | | | | | | | | | | | | |
|  | **Frequency and proportion of cases where factors that created facilitators to parental consent for organ donation were identified** | | | | | | | | | | | | | | | | | | | | |
|  | **Under 1 years old** | | | | | | **1 – 10 years of age** | | | | | | **11 – 15 years of age** | | | | **16 – 17 years of age** | | | | |
|  | **Parents who did not give consent to organ donation (n = 71)** | | **Parents who did give consent to organ donation (n = 31)** | **Total (n = 102)** | | | **Parents who did not give consent to organ donation (n = 109)** | **Parents who did give consent to organ donation (n = 89)** | | **Total (n = 198)** | | | **Parents who did not give consent to organ donation (n = 77)** | **Parents who did give consent to organ donation (n = 83)** | | **Total (n = 160)** | **Parents who did not give consent to organ donation (n = 45)** | | **Parents who did give consent to organ donation (n = 89)** | | **Total (n = 134)** |
| Parents raised organ donation as an option or had already thought about organ donation prior to approach. | 4 (6%) | | 8 (26%) | 12 (12%) | | | 3 (3%) | 23 (26%) | | 26 (13%) | | | 2 (3%) | 12 (14%) | | 14 (9%) | 2 (4%) | | 21 (24%) | | 23 (17%) |
| Altruism | 0 (0%) | | 7 (23%) | 7 (7%) | | | 0 (0%) | 26 (%) | | 26 (13%) | | | 0 (0%) | 24 (29%) | | 24 (15%) | 0 (0%) | | 19 (21%) | | 19 (14%) |
| Something positive | 0 (0%) | | 4 (13%) | 4 (4%) | | | 0 (0%) | 11 (%) | | 11 (6%) | | | 0 (0%) | 13 (16%) | | 13 (8%) | 0 (0%) | | 3 (3%) | | 3 (2%) |
| Parents felt that their child would have wanted to donate | 0 (0%) | | 0 (0%) | 0 (0%) | | | 0 (0%) | 6 (%) | | 6 (3%) | | | 0 (0%) | 11 (13%) | | 11 (7%) | 0 (0%) | | 11 (12%) | | 11 (8%) |
| Child expressed or recorded a choice to donate | 0 (0%) | | 0 (0%) | 0 (0%) | | | 0 (0%) | 2 (%) | | 2 (1%) | | | 0 (0%) | 6 (7%) | | 6 (4%) | 0 (0%) | | 15 (17%) | | 15 (11%) |
| General support for organ donation (no mention for reasons why) | 0 (0%) | | 4 (13%) | 4 (4%) | | | 0 (0%) | 5 (%) | | 5 (3%) | | | 0 (0%) | 5 (6%) | | 5 (3%) | 0 (0%) | | 3 (3%) | | 3 (2%) |
| Child or family member previous or potential beneficiary of organ donation | 0 (0%) | | 3 (10%) | 3 (3%) | | | 0 (0%) | 9 (%) | | 9 (%) | | | 0 (0%) | 0 (0%) | | 0 (0%) | 0 (0%) | | 3 (3%) | | 3 (2%) |
| Child’s personality | 0 (0%) | | 0 (0%) | 0 (0%) | | | 0 (0%) | 4 (%) | | 4 (5%) | | | 0 (0%) | 2 (2%) | | 2 (1%) | 0 (0%) | | 6 (7%) | | 6 (4%) |
| Pride and legacy through organ donation | 0 (0%) | | 0 (0%) | 0 (0%) | | | 0 (0%) | 4 (%) | | 4 (5%) | | | 0 (0%) | 3 (4%) | | 3 (2%) | 0 (0%) | | 2 (2%) | | 2 (1%) |

*Data displayed as frequency of cases (absolute numbers) and proportion of cases (%) where factors creating barriers or facilitators to parental consent for organ donation were identified, broken down by whether a parental consented and the child age group. The proportion of cases where a barrier/facilitator occurred was calculated as the frequency of barrier/facilitator divided by the group size in each category.*
